# Herbicide Selection Promotes Antibiotic Resistance in Soil Microbiomes

**DOI:** 10.1093/molbev/msab029

**Published:** 2021-02-16

**Authors:** Hanpeng Liao, Xi Li, Qiue Yang, Yudan Bai, Peng Cui, Chang Wen, Chen Liu, Zhi Chen, Jiahuan Tang, Jiangang Che, Zhen Yu, Stefan Geisen, Shungui Zhou, Ville-Petri Friman, Yong-Guan Zhu

**Affiliations:** 1 Fujian Provincial Key Laboratory of Soil Environmental Health and Regulation, College of Resources and Environment, Fujian Agriculture and Forestry University, Fuzhou 350002, China; 2 Guangdong Key Laboratory of Integrated Agro-environmental Pollution Control and Management, Guangdong Institute of Eco-environmental Science & Technology, Guangzhou 510650, China; 3 Laboratory of Nematology, Wageningen University, Wageningen 6700AA, Netherlands; 4 Department of Biology, University of York, Wentworth Way, York YO10 5DD, United Kingdom; 5 Key Lab of Urban Environment and Health, Institute of Urban Environment, Chinese Academy of Sciences, Xiamen 361021, China

**Keywords:** agrochemicals, antibiotic resistance, experimental evolution, herbicide tolerance, soil microbiomes, mobile genetic elements

## Abstract

Herbicides are one of the most widely used chemicals in agriculture. While they are known to be harmful to nontarget organisms, the effects of herbicides on the composition and functioning of soil microbial communities remain unclear. Here we show that application of three widely used herbicides—glyphosate, glufosinate, and dicamba—increase the prevalence of antibiotic resistance genes (ARGs) and mobile genetic elements (MGEs) in soil microbiomes without clear changes in the abundance, diversity and composition of bacterial communities. Mechanistically, these results could be explained by a positive selection for more tolerant genotypes that acquired several mutations in previously well-characterized herbicide and ARGs. Moreover, herbicide exposure increased cell membrane permeability and conjugation frequency of multidrug resistance plasmids, promoting ARG movement between bacteria. A similar pattern was found in agricultural soils across 11 provinces in China, where herbicide application, and the levels of glyphosate residues in soils, were associated with increased ARG and MGE abundances relative to herbicide-free control sites. Together, our results show that herbicide application can enrich ARGs and MGEs by changing the genetic composition of soil microbiomes, potentially contributing to the global antimicrobial resistance problem in agricultural environments.

## Introduction

Herbicides, such as glyphosate, dicamba, and glufosinate, are widely used to control weeds in agriculture (Maggi et al. 2019). While these compounds are thought to be specific to plants, they can cause collateral damage to other types of organisms by targeting evolutionarily conserved pathways (Motta et al. 2018; Leino et al. 2020). For example, the molecular target of glyphosate—the shikimate pathway—is found in bacteria and fungi and is crucial for the synthesis of three essential amino acids (phenylalanine, tyrosine, and tryptophan) (Motta et al. 2018; Brilisauer et al. 2019). Previous studies have linked herbicide use to compositional changes in host-associated microbiomes in insects (Motta et al. 2018), mice (Wang, Berdy et al. 2020), and plants (Hu et al. 2019), which could also potentially change the ecology of these microbial communities and how they interact with their host species. However, the potential evolutionary consequences of herbicide selection for the genetic composition and functioning of bacterial communities are poorly understood in soil microbiomes (Hu et al. 2019; Rainio et al. 2021).

Repeated herbicide exposure during weed control could select for increased herbicide tolerance in bacteria (Rainio et al. 2021). Bacterial tolerance to herbicides is known to vary both between and within species (Tothova et al. 2010; Bote et al. 2019) and could be achieved via genetic changes in the herbicide target gene (Wicke et al. 2019) or nontarget genes linked with generalized stress tolerance (Staub et al. 2012; Comont et al. 2020). Several studies have demonstrated a positive correlation between herbicide use and antibiotic resistance (Kurenbach et al. 2015,^2017,2018^; Xu et al. 2019), suggesting that herbicides could indirectly drive antibiotic resistance evolution. Mechanistically, this relationship could be explained by changes in herbicide-mediated gene expression, leading to activation of bacterial antibiotic resistance genes (ARGs) (Staub et al. 2012). For example, increased efflux pump activity has previously been shown to increase *Escherichia coli* resistance to glyphosate (Staub et al. 2012) and affect the antibiotic sensitivity of *E. coli* and *Salmonella typhimurium* bacteria (Kurenbach et al. 2015). Alternatively, long-term herbicide exposure could promote antibiotic resistance indirectly via cross-resistance evolution if the same mutations increase bacterial tolerance to both herbicides and antibiotics (Comont et al. 2020). As most current evidence is based on simplified lab experiments using single bacterial strain, more ecologically realistic experiments are required to understand herbicide selection for antimicrobial resistance in complex soil microbial communities.

Here we used direct selection experiments in soil microcosms combined with genome sequencing and transcriptomics to causally address the ecological and evolutionary effects of three widely used herbicides (Maggi et al. 2019)(glyphosate, glufosinate, and dicamba) in soil bacterial communities. We then used environmental sampling across 11 provinces across China to investigate if the observed patterns held at wider geographical scale. Our findings provide compelling evidence how herbicide selection can enrich ARGs in agricultural environments, by changing the genetic composition of bacterial populations via rapid de novo evolution of resistant genotypes, and promoting horizontal gene transfer of multidrug resistance plasmids. The role of herbicides in global antibiotic resistance problem should thus be re-evaluated, to better understand associated risks for the prevalence of ARGs in agricultural environments where soil microbiota is repeatedly exposed to herbicides during weed control.

## Results

### Herbicides Had Very Small Effects on the Abundance and Composition of Soil Microbiota

**Fig. 1.**
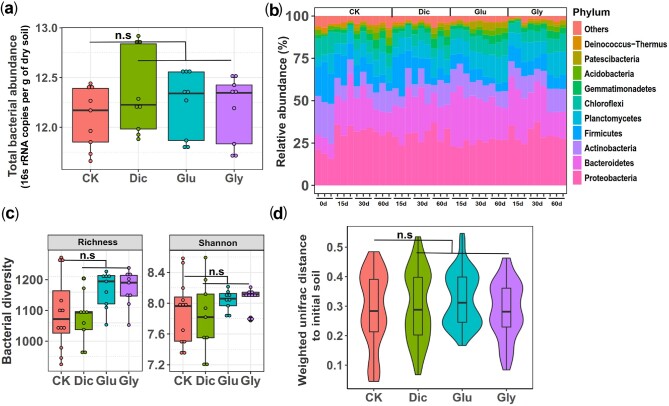
The effect of herbicide exposure on bacterial abundances, diversity, and community composition during soil microcosm experiment based on 16S rRNA amplicon sequencing. (*a*) The total bacterial abundances (16S rRNA copies per g of dry soil) between control (CK), dicamba (Dic), glufosinate (Glu), and glyphosate (Gly) herbicide treatments at the end of the soil microcosm experiment (*n* = 9 biological replicates per treatment). (*b*) The relative bacterial abundances at the phyla level between control and herbicide treatments at the beginning (0 d), 15 d, 30 d, and 60 d after initial herbicide application. (*c*) Changes in bacterial community species richness (right) and alpha diversity (Shannon index; left) between control and herbicide treatments (*n* = 9 and *n* = 12 biological replicates for herbicide and control treatments, respectively). (*d*) Changes in community composition in terms of weighted-Unifrac distances between control and herbicide treatments relative to the initial soil sample. In (*a, c, d*), box and violin plots encompass 25th–75th percentiles, whiskers show the minimum and maximum values, and the midline shows the median (dots present biologically independent replicates and ‘n.s’ denotes for nonsignificant difference [*P *≥* *0.05]). The significant differences between control and herbicide treatments were analyzed using Wilcoxon signed-rank test.

We first studied the effects of herbicides on bacterial communities and relative ARG and mobile genetic elements (MGEs) abundances in a 60-day-long soil microcosm experiment with an initial application of 10 mg of herbicides per kg of soil. Despite a continuous trend of herbicide degradation, 18–34% of herbicide residues were still detectable after 60 days of the initial application (82.92% of glyphosate, 79.12% of glufosinate, and 65.97% of dicamba degraded, Supplementary fig. 1). The herbicide-mediated effects on the abundances and composition of soil bacterial communities were studied at different time points (0, 15, 30, and 60 days) using 16S rRNA amplicon sequencing. None of the herbicides had significant effects on total bacterial abundances at the end of the experiment (*F*_3,8_ =* *1.770, *P *=* *0.6213; fig. 1*a*). While glufosinate significantly changed the relative abundances of Bacteroidetes, Planctomycetes, Chloroflexi, and Proteobacteria (fig. 1*b* and Supplementary Fig. 2*a*, *P *<* *0.001, 60 d exposure), dicamba and glyphosate had no effects on the dominant bacterial phyla (accounting for 96.4% of total sequences, *P *>* *0.05,Fig. 1*b* and Supplementary fig. 2*b* and *c*). Moreover, none of the herbicides affected bacterial community richness, alpha diversity (fig. 1*c*,*P *>* *0.05) or composition (fig. 1*d* and Supplementary fig. 3, Adonis test,*P *>* *0.05). Together, these results suggest that herbicides had minor effects on the abundance and composition of the soil microbiomes.

### Herbicide Selection Increases the Abundance of Genes Related to Antibiotic Resistance and Mobile Genetic Elements

**Fig. 2.**
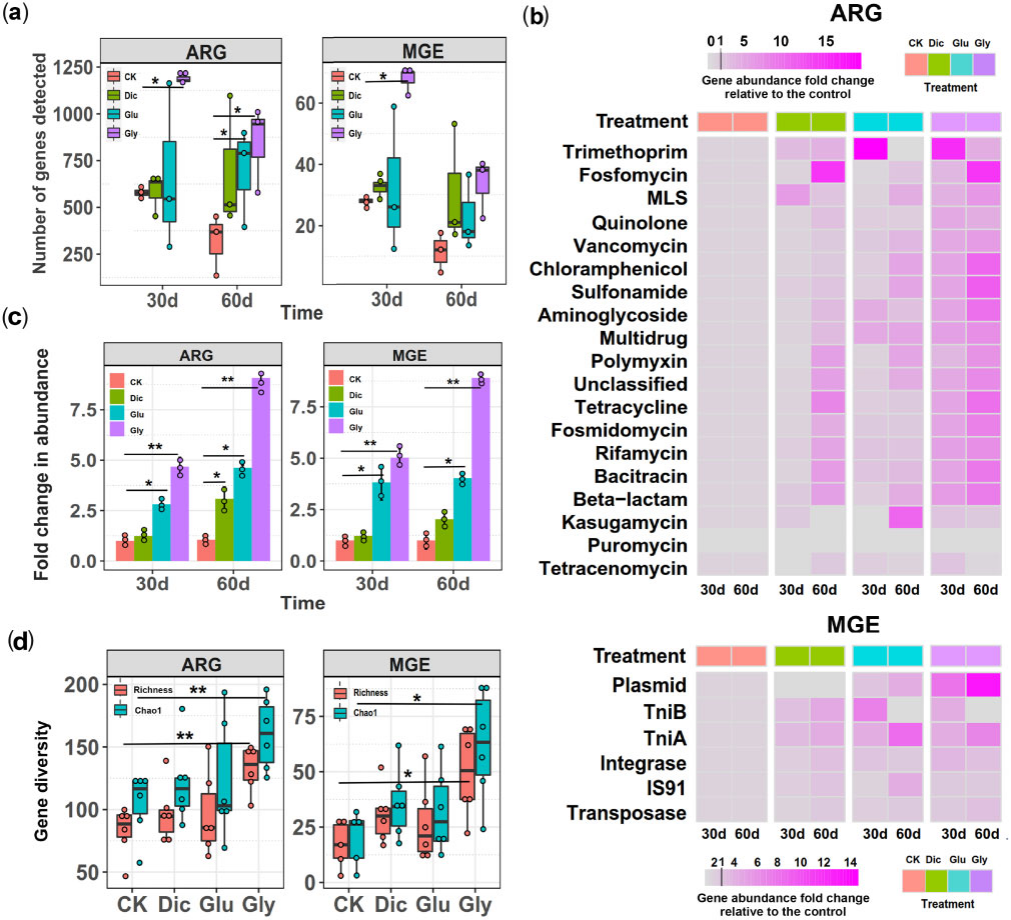
Changes in the abundance, composition and diversity of ARGs and MGEs during the soil microcosm experiment based on metagenomic sequencing. (*a*) Changes in the number of detected ARGs and MGEs in different treatments after 30 and 60 days of initial herbicide application. (*b*) Heatmaps representing the mean abundance fold changes of individual ARGs (upper panel) and MGEs (lower panel) between different treatments relative to initial soil sample (values below 1 denote for a reduction and values above 1 increase in gene abundances). (*c*) The fold changes in the total ARG (left) and MGE (right) abundances in control (CK), dicamba (Dic), glufosinate (Glu), and glyphosate (Gly) treatments after 30 and 60 days of initial herbicide application. (*d*) The diversity of ARGs and MGEs in different treatments. In (*a–c*), data shows mean ± SD of three biological replicates per treatment at each time point (*n* = 3). In (*d*), data is averaged over both sampling time points showing mean ± SD of six biological replicates per treatment. In (*a*, *c* and *d*), box plots encompass 25th–75th percentiles, whiskers show the minimum and maximum values, and the midline shows the median (dots present biologically independent replicates and asterisks denote for significant differences [*P <* *0.05, **P <* *0.01]). Wilcoxon signed-rank test was used to analyze differences between control and herbicide treatments at both 30 d and 60 d time points.

Metagenomic sequencing was applied to determine herbicide effects on the relative ARG and MGE abundances 30 and 60 days after initial application. The number of detected ARGs (*F*_3,8_* *=* *6.561,*P *=* *0.0253) and MGEs (*F*_3,8_* *=* *9.229, *P *=* *0.0115) increased over 2-fold compared to control treatment under glyphosate herbicide exposure (fig. 2*a*, 30 d exposure). In total, 22 ARG types and 304 ARG subtypes (including 3,686 nonredundant ARG genes, Supplementary table S1) were detected (average 0.3576 ARG copies per cell), which were associated to sulfonamide (28.2%), multidrug (16.6%), chloramphenicol (15.4%), tetracycline (13.0%), vancomycin (6.6%), and aminoglycoside (5.5%) resistances. The total ARG abundances significantly increased both in glyphosate (*F*_3,8_* *= 63.245, *P *<* *0.001) and glufosinate treatments (*F*_3,8_* *= 43.986, *P *<* *0.001, 30 d exposure) with glyphosate showing 9-fold increase at the end of the experiment (*F*_3,8_ = 43.986, *P *<* *0.001, 60 d exposure, fig. 2*b* and *c*). Specifically, glyphosate and glufosinate increased the abundance of aminoglycoside (*F*_3,8_ = 6.43, *P *=* *0.014) and multidrug (*F*_3,8_ = 8.15, *P *=* *0.0081) resistance genes (30 d exposure, Supplementary fig. 4), while dicamba significantly enriched the ARG subtypes linked with aminoglycoside (*F*_3,8_ = 71.87, *P *<* *0.0001) and tetracycline (*F*_3,8_ = 41.56, *P *<* *0.001) resistances (60 d exposure, Supplementary fig. 5). Glyphosate had the relatively largest effect compared to other herbicides, especially increasing the trimethoprim (60 d exposure, 14-fold), kasugamycin (30 d exposure, 2-fold), and fosfomycin (60 d exposure, 14-fold) resistance gene abundances (fig. 2*b*). Multiple MGEs were detected including transposases, *IS91* family insertion sequences, integrases, plasmids, and transposable elements (*TniA* and *TniB*), and on average, 0.0263 MGE copies per bacterial cell were observed (Supplementary table S2). Both glufosinate (4-fold increase, *F*_3,8_ = 21.298, *P *=* *0.00134) and glyphosate (8-fold increase, F_3,8_ = 106.445, *P *<* *0.0001) significantly increased total MGE abundances relative to the control treatment, while dicamba had nonsignificant effects (fig. 2*b* and *c*, 60 d exposure). Specifically, transposase (*F*_3,8_ = 5.75, *P *=* *0.021), *IS91* (*F*_3,8_ = 6.52, *P *=* *0.015), and *TniA* (*F*_3,8_ = 737, *P *=* *0.011) gene abundances increased under glyphosate exposure (30 d exposure, fig. 2*b* and *c*; Supplementary fig. 6), while plasmid (*F*_3,8_ = 48.93, *P *<* *0.001) gene abundances increased 14-fold in the glyphosate treatment (60 d exposure; Supplementary fig. 7). The diversity of both ARGs and MGEs (richness and Chao1 index) showed increasing trends, and the structure of resistome and mobilome shifted under herbicide exposure (NMDS and Bray–Curtis; Adonis test, *P *<* *0.05, Supplementary fig. 8). Overall, ARG and MGE abundances were positively correlated (Supplementary fig. 9). Qualitatively similar results were obtained using quantitative PCR targeting a subset of ARGs and MGEs (for full description see Supplementary data1 and Supplementary figs. 10–12), and a clear increase in the relative abundance of culturable antibiotic-resistant bacteria across various taxa was observed at 30* *d time point (Supplementary data and Supplementary fig. 13). Together, these results show that herbicide exposure can increase the relative ARG and MGE abundances with glyphosate and glufosinate having relatively stronger effects compared to dicamba.

### Herbicides Can Select for de novo Mutants with High Levels of Antibiotic Resistance

**Fig. 3.**
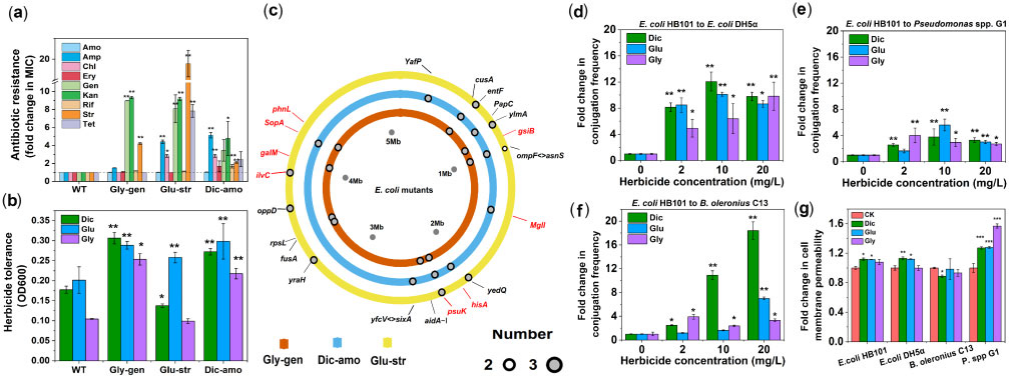
The effect of herbicide selection on the de novo antibiotic resistance evolution and horizontal gene transfer of multidrug resistance plasmid. (*a*) The MIC fold changes of evolved clones relative to ancestor (WT) clone against amoxicillin (Amo), ampicillin (Amp), chloramphenicol (Chl), gentamicin (Gen), kanamycin (Kan), tetracycline (Tet), erythromycin (Ery), rifampicin (Rif), and streptomycin (Str) antibiotics. Evolved mutants originated from glyphosate (Gly-gen), glufosinate (Glu-str), and dicamba (Dic-amo) treatments. (*b*) Herbicide tolerance measured as the growth (optical density at 600 nm) of evolved and ancestor (WT) clones in high glyphosate (500 mg/l), glufosinate (3,500 mg/l), and dicamba (100 mg/l) concentrations after 12 h of incubation. (*c*) Circular genome plot showing parallel mutations of evolved clones associated with antibiotic (black text) and herbicide (red text) tolerance. Colored circles refer to different herbicide exposure treatments, and grey and white dots the number of parallel mutations observed in clones within herbicide treatments (out of three sequenced replicates). Text on the outer circle shows the names for the mutated genes. (*d–f*) Fold changes in the conjugation frequency of RP4 multidrug resistance plasmid from *Escherichia coli* HB101 donor strain to *E. coli* DH5α (*d*), *Pseudomonas* spp. G1 (*e*) and *Bacillus oleronius* C13 (*f*) recipient strains in different herbicide concentrations. (*g*) Fold changes in cell membrane permeability in the absence (CK) and presence of glyphosate (Gly), glufosinate (Glu) and dicamba (Dic) herbicides at 10 mg/l concentration (Donor: *E. coli* HB101; Recipient: *E. coli* DH5α; *B. oleronius* C13; *Pseudomonas* spp G1). In all panels (except for *c*), data show mean ± SD of three biological replicates per treatment (*n* = 3), while asterisks denote for significant differences (*P <* *0.05, **P <* *0.01, ***P <* *0.001). Differences between treatments were analyzed using ANOVA followed by multiple comparison using Tukey HSD test.

To understand underlying mechanisms, we first tested if herbicide exposure selects for de novo antibiotic-resistant mutants. Nine different antibiotics were chosen based on the observed changes in corresponding resistance genes under herbicide selection during the soil microcosm experiment. Using experimental evolution, we exposed *E. coli* DH5α strain to all three herbicides in multiple concentrations (0, 5, 10, 20, 25, and 50 mg/l) for 30 daily serial transfers in liquid Luria-Bertani (LB) media. Even though none of the herbicide concentrations had clear effects on *E. coli* DH5α growth (Supplementary fig. 14), the frequency of bacteria with enhanced tolerance to gentamycin, streptomycin, and amoxicillin increased in all herbicide treatments (herbicide presence: *F*_3,62_ = 11.778, *P *<* *0.001; exposure time: *F*_4,59_ = 8.472, *P *<* *0.001; herbicide concentration: *F*_3,62_ = 2.326, *P *=* *0.083; Supplementary fig. 15). The frequency of bacteria with increased resistance to other antibiotics (ampicillin, chloramphenicol, kanamycin, tetracycline, erythromycin, and rifampicin) did not change in any of the herbicide treatments. Specifically, dicamba exposure enriched bacteria that had 5-fold higher antibiotic resistance compared to glyphosate and glufosinate treatments (Supplementary fig. 16). To explore this in more detail, we randomly isolated three colonies (one per replicate) that showed up to 19-fold increases in antibiotic MIC_90_ (fig. 3*a*) from all herbicide treatments (5 mg/ml concentration) using gentamycin (glyphosate treatment; labeled Gly-gen;), streptomycin (glufosinate treatment; labeled Glu-str), and amoxicillin (dicamba treatment; labeled Dic-amo) selective plates. All evolved clones showed elevated growth after 12 h incubation in high dicamba (100 mg/l), glyphosate (500 mg/l), and glufosinate (3,500 mg/l) concentrations relative to the ancestral strain, indicative of increased herbicide tolerance (fig. 3*b* and Supplementary table S3). To understand the molecular basis of herbicide and antibiotics tolerance evolution, all clones were genome sequenced. Majority of the variants had nonsynonymous SNPs with predicted changes in encoding proteins (Supplementary table S4). Glyphosate-exposed clones had unique mutations in *rspL* (30S ribosomal protein)*, fusA* (Elongation factor G), *aidA-I* (AIDA-I autotransporter), and *cusA* (Cation efflux system protein) genes, which have previously been linked to resistance against aminoglycosides (streptomycin and ampicillin) (Fischer et al. 1977; Carr et al. 2006; Ibacache-Quiroga et al. 2018), aminopenicillins (Wells et al. 2007), and heavy metals (Tanaka et al. 2017) (fig. 3*c* and table 1). In contrast, dicamba-exposed clones had unique mutations in *YafP* (Putative N-acetyltransferase) and *PapC* (Outer membrane protein) genes and intergenic region between *yfcV* (Fimbrial protein) and *sixA* (Phosphohistidine phosphatase) genes known to confer resistance to aminoglycoside (gentamicin) and β-lactam (ampicillin) antibiotics (Avalos Vizcarra et al. 2016; Favrot et al. 2016; May and Grabowicz 2018) (fig. 3*c* and table 1). Clones that were exposed to glufosinate had unique mutations in *oppD* (Oligopeptide transport ATP-binding protein), *yraH* (Putative fimbrial-like protein), and *yedQ* (Putative diguanylate cyclase) genes, associated with resistance to various aminoglycosides, macrolides, polymyxins, and fluoroquinolones (Gupta et al. 2014; Avalos Vizcarra et al. 2016; Locher 2016) (fig. 3*c* and table 1). Moreover, eight out of nine clones had intergenic mutation between *ompF* (Outer membrane porin F) and *asnS* (Asparagine-tRNA ligase) genes, which have been linked to carbapenem and cefepime resistances (Zheng and Nolan 2014; Ghai I and Ghai S 2018).

**Table 1.** The Genetic Mutations Linked with Antibiotic (Black Text) and Herbicide (Red Text) Tolerance for Nine Clones Evolved in Different Herbicide Exposure Treatments.This color represents the mutation of the corresponding bacteria

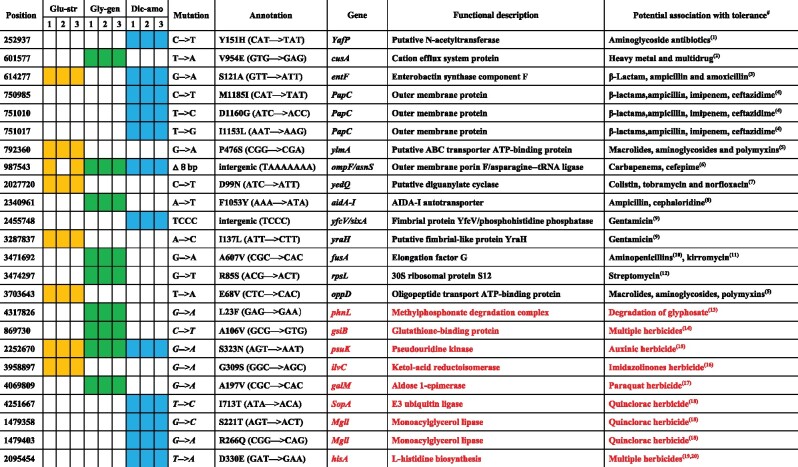

Note: # denotes for references listed in supplementary file. The color of cells represents the mutation of genes in the corresponding strain.

Multiple candidate mutations linked to herbicide tolerance were also found (fig. 3*c* and table 1). For example, mutations in *phnL* (Methylphosphonate degradation complex; linked to glyphosate metabolism), *gsiB* (Glutathione-binding protein; linked to glutamate metabolism), and *galM* (Aldose 1-epimerase; linked to stress tolerance) were observed in glyphosate-exposed clones (Otoshi et al. 2001; Cummins et al. 2013; Wang et al. 2016) (fig. 3*c* and table 1). Moreover, mutations in *ilvC* and *psuK* (Ketol-acid reductoisomerase and pseudouridine kinase; linked to herbicide target proteins) genes were found in glufosinate-exposed clones (Abell 1996; Delye et al. 2015) (fig. 3*c* and table 1), and mutations in four genes (*psuK*, *SopA*, *Mgll*, and *hisA;* linked to herbicide target protein and L-histidine biosynthesis, respectively) were observed in dicamba-exposed clones (Ohta et al. 1997; Christoffoleti et al. 2015; Dahabiyeh et al. 2019) (fig. 3*c* and table 1). We also found that even short-term herbicide exposure (6 h) led to increased expression of efflux pumps, transporters, and glutamate metabolism with the ancestral clone (Supplementary fig. 17*a*), which are all known to increase tolerance to xenobiotics such as antibiotics and herbicides (Conte and Lloyd 2011; Cummins et al. 2013). Together, these results suggest that herbicides can promote antibiotic tolerance by changing bacterial gene expression, or by selecting for more tolerant mutants de novo during prolonged herbicide exposure.

### Herbicides Promote the Horizontal Transfer of Multidrug Resistance Plasmid

Due to positive correlations between ARGs and MGEs in the soil microcosm experiment, we tested if herbicide exposure could promote horizontal gene transfer of ARGs by quantifying conjugation frequency of RP4 multidrug resistance plasmid within (from *E. coli* HB101 to *E. coli*DH5α) and between bacterial species (from to from *E. coli* HB101 to *Pseudomonas* spp. G1 or *Bacillus oleronius* C13). The presence of herbicides and exposure concentration significantly increased conjugation frequency independent of the herbicide type (Supplementary table S5; the presence of RP4 plasmid in conjugants confirmed with PCR, Supplementary fig. 18). While the conjugation frequency varied between bacterial species (*F*_2,105_ = 5.714, *P* = 0.00441), it was generally highest within compared to between species (from *E. coli* DH5α to *E. coli* HB101, fig. 3*d*–*f*). The observed pattern could potentially be explained by chemical stress, which is known to promote plasmid conjugation due to increased cell membrane permeability (Qiu et al. 2012; Wang et al. 2019). In support for this, we found that bacterial cell membrane permeability increased after 6 h exposure to all herbicides (10 mg/l; *F*_3,8_ = 48.34, *P *<* *0.001; except for *B. oleronius* C13, fig. 3*h*). In the case of the *E. coli* HB101 donor strain, the outer membrane protein (*ompW*) and inner membrane protein (*yhiD*) genes were significantly upregulated upon exposure to dicamba by 3.1 and 4.9-fold, respectively (Supplementary fig. 17*a*), which suggests that these proteins could be important for initiating plasmid transfer. Transmission electron microscopy was further used to confirm that all herbicides severely damaged the cell membranes of all bacteria (Supplementary fig. 19). Another alternative explanation for the increased conjugation frequency could be the accumulation of intracellular reactive oxygen species (ROS) and SOS response, which can lead to oxidative damage to DNA, cell components, and cell membranes (Qiu et al. 2012), but also increase the frequency of conjugation (Wang et al. 2019). To counteract this damage, bacteria often upregulate the production of antioxidant enzymes, such as catalase (CAT) and superoxide dismutase (SOD), which are known to protect them from ROS and SOS. In contrast with some previous studies (Qiu et al. 2012; Huang et al. 2019; Wang et al. 2019), our transcriptomic analysis and enzyme activity found no clear change of these antioxidant enzymes (Supplementary fig. 20), despite a few genes (*sodB* and *ygiW*) that were upregulated during dicamba exposure (Supplementary fig. 17*b*). The lack of the potential role of ROS was verified by measuring conjugation frequency in the presence of herbicides supplemented with the ROS scavenger thiourea, which removes the potential benefits of ROS for conjugation. Thiourea had only a small effect on conjugation frequency under glyphosate exposure (Supplementary fig. 21), which confirms that ROS was not the main driver of herbicide-induced increase in conjugation frequency. However, herbicide exposure significantly increased the expression of 13 glutamate metabolism genes (Supplementary fig. 17*a*) that have previously been associated with other stress responses (Feehily and Karatzas 2013). It is thus possible that upregulation of these genes was linked to increased herbicide tolerance. Together, these results indicate that instead of ROS and SOS, increased cell membrane permeability and upregulation of other stress-related genes were more important for the increased conjugation frequency under herbicide exposure.

### Herbicide Application History Correlates with Relatively Higher ARG and MGE Levels in Agricultural Soils in China

**Fig. 4.**
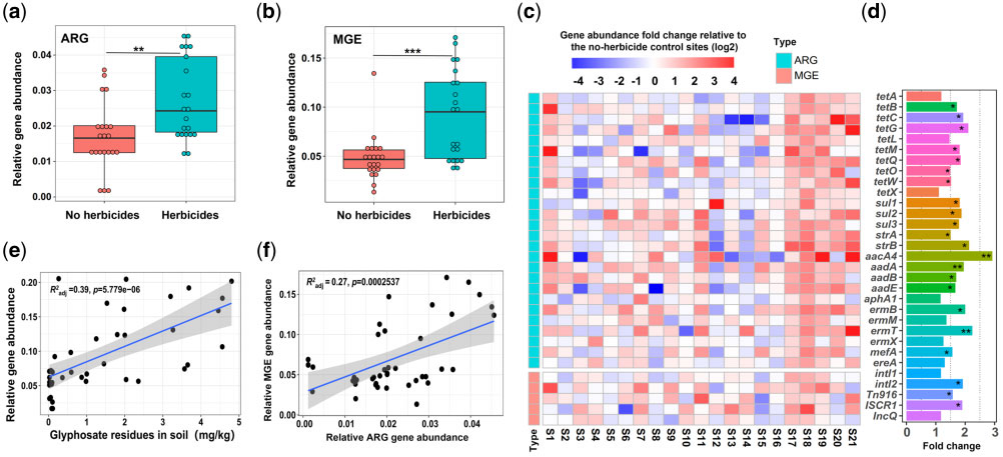
The effects of glyphosate herbicide application on ARG and MGE abundances across 21 sampled field sites in China based on qPCR analysis of selected target genes. (*a, b*) The relative ARG and MGE abundances in fields with contrasting herbicide use histories. (*c*) Heatmaps representing fold changes of individual ARG (upper panel) and MGE (lower panel) abundances relative to herbicide-free control sites (S1-21 on X-axis denotes for 21 sampling sites). (*d*) The mean target gene abundances across all the sampled herbicide-sites shown as fold changes relative to herbicide-free control sites. (*e*) Positive correlation between relative ARG and MGE abundances and detected glyphosate residues in the sampled soils. (*f*) Positive correlation between relative MGE and ARG abundances across all sampled sites. The shaded area depicts the 95% confidence interval and R2 the adjusted coefficient of determination with the slope of the best-fit trendlines. In (*a, b, e, f*) data is based on 42 biological replicates (*n* = 21 for each group) and data in plot (*c*) shows the mean values based on three technical replicates per site (*n* = 3). In (*a, b, d*), asterisks denote for significant differences based on Wilcoxon signed-rank test (*P <* *0.05, **P <* *0.01, ***P <* *0.001).

To explore potential implication of herbicide application history in agricultural fields, we sampled a total of 21 sites across 11 provinces in China (Supplementary fig. 22), and compared the relative abundances of ARGs and MGEs between fields that had been 1) continuously exposed to glyphosate (at least for 10 years) or 2) had been free of any herbicide use for the past 5 years. Based on qPCR analyses targeting a subset of ARGs and MGEs (Supplementary table S6), we found that herbicide application was associated with higher relative abundances of both ARGs and MGEs (*P *<* *0.01, fig. 4*a*–*c*). Specifically, high abundances ofthe *aacA4* aminoglycoside resistance gene were observed both in the soil microcosm experiment and field samples (fig. 4*d*, Supplementary fig. 23). Furthermore, while observed relationships were sensitive to individual sites and specific target genes (fig. 4*c* and *d*), ARG and MGE abundances showed positive correlations with detected glyphosate residues in the soil (fig. 4*e*). Similar to the soil microcosm experiment, total ARG and MGE abundances correlated positively with each other (fig. 4*f*), suggesting that horizontal gene transfer could be an important mechanism for herbicide-mediated ARG enrichment in agricultural soil microbiomes.

## Discussion

Together, our multimethodological approach shows that herbicide exposure can increase the prevalence of ARGs and MGEs in soil microbiomes. Herbicides persisted in soil microcosms throughout the experiment, and while their concentrations declined gradually after the initial application, the abundance of multiple ARGs and MGEs increased steadily over time. Glyphosate had the relatively largest effect compared to other herbicides, especially increasing the abundance of trimethoprim and kasugamycin resistance genes and plasmid, integrase and transposase MGEs. In contrast, glufosinate increased the abundance of kasugamycin, tetracycline, and plasmid genes, while dicamba increased the abundance of trimethoprim and *TniA* transposase genes. This pattern of elevated resistance could be explained by two potential underlying mechanisms: selection for de novo antibiotic resistance and mobilization of ARGs via horizontal gene transfer. Herbicide exposure selected for mutations linked to detoxification of xenobiotics (e.g., efflux pumps, glyphosate and amino acid metabolism, target genes and stress tolerance) (Abell 1996; Ohta et al. 1997; Otoshi et al. 2001; Conte and Lloyd 2011; Cummins et al. 2013; Christoffoleti et al. 2015; Delye et al. 2015) and several antibiotic resistance mechanisms: efflux pumps and transporters (Hall and Mah 2017), changes in the antibiotic target genes (ribosomal proteins) (Suzuki et al. 2014) and degradation or enzymatic modification of antibiotics (hydrolytic enzymes) (Zampieri et al. 2017). Efflux pumps have previously been linked to both antibiotic and herbicide resistances (Paul et al. 2019) and could have provided cross-resistance to both types of compounds (Xing et al. 2020). However, most of the mutations were unique to specific herbicide treatments, indicative of distinct tolerance mechanisms potentially driven by different modes of action of herbicides (Sherwani et al. 2015; Comont et al. 2020). Also, multiple different resistance mutations were found in every clone, which suggests that potential synergistic pleiotropic effects of resistance should be explored in the future.

Herbicides could have promoted the de novo resistance evolution by increasing bacterial mutation frequency leading to increase in genetic variation for the selection to act on. While this explanation is not supported by a previous study where the genome-wide mutation rate of *E. coli* was found to decrease with increasing glyphosate-based herbicide concentrations (Tincher et al. 2017), it warrants more detailed testing in the future. Alternatively, the resistance mutations could have been slightly beneficial when in competition with nonresistant ancestral genotypes at sublethal herbicide concentrations similar to selection of antibiotic resistance at very low antibiotic concentrations (Gullberg et al. 2011). Furthermore, herbicides mobilized ARGs by increasing RP4 plasmid conjugation frequency, which is a broad-host-range conjugative IncP α-type plasmid and can transfer between 15 different bacterial phyla (Fan et al. 2019). Based on our experiments, the most likely explanation behind this result was herbicide-induced increase in bacterial cell membrane permeability (Wang et al. 2019), which promoted plasmid movement. While it was confirmed that this was not driven by increased expression of SOS or ROS related genes, upregulation of glutamate metabolism genes that have previously been associated with acid and other stress tolerance was observed. More detailed molecular studies are still required to better understand the effects of herbicides for plasmid conjugation frequency.

Together, the observed mechanisms could have changed the relative ARG and MGE abundances by acting on intraspecies genetic variation, and by promoting the spread of ARGs along with MGEs in the bacterial community (Wang et al. 2019; Xing et al. 2020), without clear effects on the taxonomic composition of soil microbiomes often observed along with ecological species sorting. Our findings thus suggest that herbicide-mediated selection could be cryptic leading to changes in the genetic composition of bacterial populations without clear changes in species abundances (Yoshida et al. 2007). As glyphosate had clear effects in our experiments, and is one of the main herbicides used in China (Zhang et al. 2015), we compared its concentration and ARG and MGE abundances between multiple field sites in China. We found that glyphosate application history was associated with relatively higher ARG and MGE abundances compared to herbicide-free control sites in across all agricultural fields. Considerable variation was however observed between sampling sites, and hence, more work is needed to understand to what extent herbicide-mediated effects on the ARGs and MGEs depend on the physicochemical soil properties, crop species, and agricultural practices. Moreover, as no data on agrochemical application history at the field level was available, we cannot exclude the potential confounding effects caused by other types of herbicides or agrochemicals.

We conclude highlighting the potential risks of herbicides for the evolution and spread of antibiotic resistance along with other nonantimicrobial xenobiotics (Qiu et al. 2012; Lu et al. 2018; Wang, Lu et al. 2020) and chemicals (Wang et al. 2019; Wang, Chen et al. 2020). For example, herbicide-induced mobilization of ARGs could potentially relocalize resistance genes from harmless soil bacteria to human opportunistic pathogens, especially when fields are fertilized with untreated manure that typically contains both ARGs and pathogenic bacterial species (Udikovic-Kolic et al. 2014). Moreover, it is important to revisit how the safe use of herbicides is assessed. For example, quantifying herbicide effects on bacterial growth in simple laboratory assays is unlikely to predict the outcomes in bacterial communities, where relatively low sublethal herbicide concentrations can change the abundance and movement of ARGs. In light of our findings, we suggest that herbicide effects on soil microbiomes should be re-evaluated to understand better the risks associated with the spread of antimicrobial resistance across agricultural environments.

## Materials and Methods

### Soil Microcosm Experiment

#### Herbicides, Antibiotics and Collection of Soil Samples

Standard chemicals for three herbicides (glyphosate, glufosinate, and dicamba) and antibiotics were acquired from Sigma-Aldrich (Germany) with a purity higher than 99.0%. Soil samples were collected from a representative vegetable cultivation base in Fujian Agriculture and Forestry University (N26°05′, E119°14′). All samples (from five fields) were collected at a depth of 0 to 15 cm, sieved (2 mm) to remove stones and debris and placed in an artificial climate room (25°C) for two weeks for stabilization before the experiments. The detailed physicochemical soil properties were measured as described previously (Yang et al. 2018) and are summarized in Supplementary table S7.

#### The Design of the Soil Microcosm Herbicide Exposure Experiment

The prestabilized soil samples were homogenized and mixed, after 150 g of mixed dry soil was transferred into individual glass bottles (150 ml volume). Soil microcosms were then exposed to three herbicide treatments (a final concentration of 10 mg/kg of glyphosate, glufosinate, and dicamba dissolved in Milli-Q water [18 MΩ·cm; Millipore, Billerica, MA, USA]), while control treatment received only the solvent (Milli-Q water). Herbicide concentrations were chosen based on residual quantity of these herbicides that typically vary between 10.19 and 33.03 mg/kg of soil(Voos et al. 1994; Dennis et al. 2018). The soil water content was adjusted to 60% of the maximum water holding capacity using sterile deionized water after all the microcosms were covered with caps equipped with breathable film. The microcosms were then transferred into a climatic chamber, arranged randomly and incubated at 30°C in the dark. Soil water contents were maintained by adding deionized water at every 2 days. Each treatment was replicated 12 times and one replicate per treatment was destructively sampled at the beginning (day 0) and 15, 30, and 60 days after the start of the experiment (chosen randomly). Destructive sampling was chosen to avoid disrupting the soil and microbiome structure in contrast to repeated sampling, which would have required throughout homogenization of the soil. All samples were stored at −20°C to later quantify residual herbicides and to extract total DNA for bacterial community analyses as described below.

#### Extraction and Determination of Residual Herbicides in Soil Microcosm Experiment

We used previously developed methods to analyze glyphosate, glufosinate, and dicamba residues at 15, 30, and 60 d during the soil microcosm experiment (Druart et al. 2011; Valle et al. 2019). After thorough homogenization of microcosm contents, independent soil samples (1.0 g) were mixed with Millipore water (10 ml), sonicated for 1 h, shaken at 25°C for 1 h and finally centrifuged at 5,000×g for 15 min. The supernatants were filtered (0.45 µm) to remove solid particles and evaporated in the freeze dryer. The residues of herbicides were dissolved in 0.5 ml of methanol (1%) for further following analysis. The concentrations of glyphosate and glufosinate were detected as described previously using precolumn derivatization followed by high-performance liquid chromatography (HPLC) on C18 column (Druart et al. 2011). The concentrations of dicamba was measured directly using HPLC with UV detector. The average recoveries (*n* = 3) for all the herbicides ranged between 80.5% and 94.3% and a more detailed description of the analytical methods is included in Supplementary materials (Supplementary Text 1).

#### DNA Extraction, Illumina Sequencing and Data Analysis of Bacterial Communities

Total genomic DNA of soil samples was extracted using a Fast DNA spin kit (MP Biomedicals, Cleveland, OH, USA) following manufacturer’s instructions. The changes in bacterial community composition and diversity were determined using 16S rRNA gene amplicon sequencing using Illumina NovaSeq 6000 PE250 platform (Guangdong Magigene Biotechnology Co. Ltd, Guangzhou, China). The universalprimers 515F (5′-GTGCCAGCMGCCGCGGTAA-3′) and 907R (5′-CCGTCAATTCMTTTRAGTTT-3′) targeting the V4–V5 region of 16S rRNA gene were used. The raw 16S rRNA gene sequences were processed using QIIME 2 as follows (version 2019.7) (Bolyen et al. 2019). Raw reads were first quality-filtered (i.e., filtered, dereplicated, denoised, merged, and assessed for chimaeras) to produce amplicon sequence variants (ASVs) using DADA2 pipeline available in QIIME2 (Callahan et al. 2016). The ASVs observed at low frequencies (less than two) were removed and remaining ASVs were classified using the QIIME2 naive Bayes classifier trained on 99% operational taxonomic units from the SILVA rRNA database (v 132) (Quast et al. 2012).

#### Metagenomic Sequencing and Data Analysis

To analyze changes in ARG (resistome) and MGE (mobilome) abundances during the soil microcosm experiment, soil samples collected at 0 d (*n* = 3), 30 d (*n* = 12), and 60 d (*n* = 12) time points were subjected to metagenomic sequencing. Total DNA samples were sent to Guangdong Magigene Biotechnology Co. Ltd for library construction and shotgun metagenomic sequencing on HiSeq platform (Illumina) with 150 bp paired-end sequencing. The raw sequencing reads for each sample were independently processed for quality control using the Trimmomatic (version 0.39) (Bolger et al. 2014). Changes in resistome and mobilome were analyzed using local ARGs-OAP (v2.2) using clean reads (Yin et al. 2018). To compare the relative ARG abundances in different samples, normalization against the total cell numbers in each sample (copies/cell) was performed. The details and data analysis are shown in Supplementary materials (Supplementary Text 2). Briefly, extracted DNA from 27 samples resulted in 15 billion paired-end reads, comprising 235 Gb of DNA. The sequencing yielded an average of 58 million paired-end reads per pooled sample (Supplementary table S8).

#### Quantitative PCR to Determine Changes in ARG and MGE Abundances

To verify changes in the ARG and MGE abundances based on metagenomic analysis, quantitative PCR was used to measure the abundance of a total of 27 ARG subtypes conferring resistance to four common antibiotics (tetracycline, sulfonamide, aminoglycoside, and macrolide) and five MGEs including two integrases (*intI1*, *intI2*), two plasmids (*ISCR1*, *IncQ*), and one transposon (*Tn916*/*1545*, abbreviated *Tn916*). The total bacterial abundances in soil samples were determined using 16S rRNA gene (341F/515R). The quantification was carried out on a Light Cycler 96 system (Roche, Mannheim, Germany) as described previously (Liao et al. 2018) and all the details of the qPCR assay for all target genes (primers, annealing temperatures, reaction conditions, and amplification cycles) and results are listed in Supplementarymaterials (Supplementary Text 3).

#### Isolation of Antibiotic Resistant Bacteria during the Soil Microcosm Experiment

Isolation of culturable antibiotic-resistant bacteria was performed at the mid-point (30 d) of soil microcosm experiment as described previously (Liao et al. 2019). Briefly, 1 g of soil sample was suspended in 9 ml of phosphate-buffered solution by shaking at 200 rpm for 30 min. Samples were then serially diluted in phosphate-buffered solution and plated on LB (10 g tryptone, 5 g yeast extract, 10 g sodium chloride, and 15 g of agar in 1 l Milli-Q water; pH: 7.4) agar supplemented with one of the four antibiotics at the following concentrations: amoxicillin (32 mg/l), chloroamphenicol (16 mg/l), erythromycin (10 mg/l), and tetracycline (16 mg/l). The numbers of colony forming units (CFU) were counted after 24–48 h of incubation at 37°C using dilutions that resulted in 20–200 CFUs per plate. A total of 115 culturable antibiotic-resistant colonies (20–30 strains resistant to each antibiotic) were randomly isolated and preserved as clonal monocultures at −80°C in 50% glycerol. To identify the taxa of isolated bacterial colonies, we extracted genomic DNA from all isolates using a Bacteria DNA Kit (Tiangen, Beijing, China) following manufacturer’s instructions. The 16S ribosomal RNA (rRNA) gene was then amplified by PCR using universal primers 27F (5-AGAGTTTGATCCTGGCTCAG-3′) and 1492R (5-GGCTACCTTGTTACGACTT-3′) and PCR products sequenced by Shanghai Shengong Biotechnology Co. (Shanghai, China).

### Herbicide Effects on the Selection for de novo Herbicide and Antibiotic Resistant Mutants and the Conjugation Rate of a Multidrug Resistance Plasmid

#### Experiment 1: Determining the Frequency of de novo Antibiotic Resistant Mutants and Herbicide Resistance under Direct Herbicide Exposure

We used an experimental evolution approach to determine the frequency of emerging antibiotic-resistant *E. coli* mutants under herbicide exposure. To this end, a single colony of *E. coli* DH5α was revitalized from −80°C stock and grown in liquid LB for 12 h at 30°C to reach cell concentrations of 10^8^–10^9^ CFU/ml. To initiate the herbicide exposure experiment, 50 μl of the overnight cell suspension was inoculated into 4.95 ml of fresh liquid LB supplemented without or with different concentrations of herbicides (5, 25, and 50 mg/l of glyphosate or glufosinate and 2, 10, and 20 mg/l of dicamba). All treatments were replicated four times and cultures grown at 30°C and shaken at 150 rpm. Every 24 h, 50 μl of the homogenized cell mixture was serially transferred to a new tube containing 4.95 ml fresh liquid LB with respective concentrations of herbicides (or no herbicides in the control treatment). Serial transfers were repeated for a total of 30 transfers (30 days) equaling around 720 bacterial generations (estimated one cell division h^−1^). The frequency of resistant colonies was estimated after 1, 5, 10, 20, 25, and 30 days from the start of the experiment by plating 100 μl of each replicate sample on LB agar plates without antibiotics (total cells) and with nine different antibiotics in following concentrations (amoxicillin [64 mg/l], ampicillin [100 mg/l], chloramphenicol [16 mg/l], gentamicin [10 mg/l], kanamycin [100 mg/l], tetracycline [4 mg/l], erythromycin [15 mg/l], rifampicin [100 mg/l], and streptomycin [30 mg/l]). Plates were incubated at 30°C for 48 h after the number of colonies per replicate plate counted. The frequency of antibiotic-resistant colonies was estimated by dividing the number of antibiotic-resistant colonies with the total bacterial counts (LB-only plates). As resistance did not evolve to some of the tested antibiotics, we mainly focused on tracking down the frequencies of gentamicin, streptomycin, and amoxicillin resistant *E. coli* clones under glyphosate exposure (10 mg/l) and gentamicin and amoxicillin resistant clones under glufosinate and dicamba treatments (5 mg/l in case of both herbicides). We also determined the minimum inhibitory concentrations (MICs) for all resistant colonies against the antibiotics used for the isolation as described previously (Lu et al. 2018). Briefly, resistant bacteria were grown in LB in the absence of herbicides and antibiotics and diluted to approximately 10^5^ cfu/ml as described previously. MIC assays were then performed on 96-well microplates with a range of antibiotic concentrations diluted in LB media (5 μl of bacterial cultures, 15 μl of antibiotics [with same concentrations as above], and 130 μl of fresh LB media). Mixtures of fresh LB medium, ethanol, and sterilized water were used as blank controls as some antibiotics could be dissolved only in the ethanol (Chl, Str, Rif, and Ery), while other were dissolved in sterilized milli-Q water (Amp, Tet, Kan, and Amo). The plates were incubated at 30°C for 20 h before measuring bacterial densities as optical density (OD600 nm) using microplate reader (Infinite^®^ 200 PRO, Tecan, Swiss). The MICs of the bacterial strains were determined as the concentration of antibiotic inhibited 90% of the bacterial growth and each resistant clone was tested in triplicate.

#### Quantifying Herbicide Tolerance after the Selection Experiment

As the herbicides used in our experiments did not impose a clear bacterial mortality, it was not possible to determine a clear breakpoint, such as MIC, which was used with antibiotics. As a result, herbicide tolerance was quantified as effects on bacterial growthunder high but still sublethal herbicide concentrations (glyphosate: 500 mg/l; glufosinate: 3,500 mg/l, and dicamba: 100 mg/l) in liquid LB media (in 96-well microplates). Bacteria were incubated at 30°C for 12 h, and growth measured (OD600) every 1 h using spectrophotometer (Infinite^®^ 200 PRO, Tecan, Swiss). All experiments were performed at least in triplicate.

#### DNA Extraction and Whole-Genome DNA Sequencing of Resistant E. coli DH5α Clones

To shed light on the underlying molecular mechanisms of resistance, three evolved *E. coli* DH5α mutants (one per replicate) and three ancestor clones were selected from each herbicide treatment for whole-genome DNA sequencing (Lu et al. 2018) using gentamycin (glyphosate treatment; labeled Gly-gen;), streptomycin (glufosinate treatment; labeled Glu-str), and amoxicillin (dicamba treatment; labeled Dic-amo) selective plates. Genomic DNA was extracted using the TIANGEN Bacteria DNA extraction kit (Tiangen, Beijing, China) following manufacturer’s instructions and overnight bacterial cultures. DNA quality was analyzed using NanoDrop spectrophotometers (Thermo Fisher Scientific) and electrophoresis gel. DNA samples were submitted to Guangdong Magigene Biotechnology Co. Ltd for the sequencing on the HiSeq platform (Illumina) with 150 bp paired-end sequencing. After quality trimming, the clean reads were de novo assembled using the SPAdes (Bankevich et al. 2012) with default parameters. The whole-genome sequence data from each strain was aligned against the *E. coli* str. K-12 substr. MG1655 reference genome (Accession NC000913.3) and variant calling performed using Breseq pipeline with default parameters (https://www.barricklab.org/twiki/bin/view/Lab/ToolsBacterialGenomeResequencing) to explore single nucleotide polymorphism (SNP) and short INDELs (insertions and deletions) as described previously (Lang et al. 2013).

#### Experiment 2: Determining the Frequency of Plasmid Conjugation under Direct Herbicide Exposure

The effect of herbicides on the frequency of plasmid conjugation was measured using *E. coli* HB101 strain carrying a multidrug resistance RP4 plasmid as a donor cell (Qiu et al. 2012) (resistant to tetracycline, kanamycin, and ampicillin; kindly gifted by Professor Junwen Li and Zhigang Qiu from Tianjin Institute of Environmental & Operational Medicine, Tianjin, China). *Pseudomonas* ssp. G1 (resistant to gentamicin), *B. oleronius* C13 (resistant to chloramphenicol), and *E. coli* DH5α (resistant to chloramphenicol) were used as recipient strains. *Pseudomonas* ssp. G1 and *B. oleronius* C13 strains were isolated from the glyphosate herbicide exposed soil of the present study (showing stable resistance to gentamicin and chloramphenicol, respectively). Before the start of the experiments, both the donor and recipient strains were incubated overnight at 30°C in LB medium and shaken at 150 rpm. Bacterial cells were then collected by centrifugation at 6,000×g for 5 min at 4°C, washed (1 × PBS, pH = 7.2) and resuspended to PBS in cell densities of 1 × 10^8^ CFU/ml. The donor and recipient strains were mixed at 1:1 ratio and exposed to different concentrations of all three herbicides in factorial experimental design (2, 10, and 20 mg/l) for 6 h at 30°C using three replicates to estimate the frequency of conjugation. The six hours exposure time was chosen to minimize the emergence of potential de novo resistance mutations during the herbicide exposure and no-herbicide (Milli-Q water only) treatment was included as a control group. The same sets of conjugation experiments were conducted also in the presence of thiourea (final concentration of 100 μM), which is an ROS scavenger inactivating any ROS produced by bacteria. The frequency of transconjugants was determined based on previously described method (Wang et al. 2019) as follows. While the RP4 plasmid conferred high resistance to tetracycline, kanamycin, and ampicillin, the recipient strains were only resistant to chloramphenicol or gentamicin. As a result, the frequency of transconjugants could be estimated by plating samples on LB agar plates containing four different antibiotics and by dividing the number of transconjugants with the number of total bacterial cells in the co-culture (total colonies on LB-only plates). PCR was also used to confirm plasmid transfer as follows. Two transconjugants were randomly selected from agar plates containing all four antibiotics (per treatment) and cultured in LB broth overnight. Plasmid extractions were performed using the Invirogen Quick Plasmid Miniprep Kit (Life Technologies, USA) following the manufacturer’s instructions. Following extraction, agarose gel electrophoresis was applied to verify the presence of plasmids in each transconjugant, and PCR was performed to confirm that the transconjugants contained the same genes as the original RP4 plasmid (Supplementary Text 4).

#### Measurement of Activity of Reactive Oxygen Species (ROS), Antioxidant Enzymes, and Cell Membrane Permeability

To mechanistically understand how herbicide exposure could affect the rate of conjugation, we measured changes in cell membrane permeability, antioxidant enzyme, and ROS activity in the absence and presence of herbicides for all recipient and donor strains. Bacterial ROS were detected with a DCFDA/H2DCFDA-cellular ROS detection assay kit (ab113851, Abcam, UK) using multimode microplate reader (Varioskan LUX2,Thermo Fisher Scientific, USA). Bacterial antioxidant enzymes, including catalase (CAT) and superoxide dismutase (SOD) activity were assayed with commercial kits manufactured by the Nanjing Jiancheng Bioengineering Institute (Nanjing, China) and a microplate reader (Infinite^®^ 200 PRO, Tecan, Swiss). The hydrolysis rate of the o-nitrophenyl-β-D-galactopyranoside (ONPG) by cell was measured to assess cell permeability in terms of absorbance values at 420 nm as described in a previous study (Jin et al. 2020). Transmission electron microscope (TEM) was performed to characterize whether the cell morphology or membranes were changed under exposure of herbicide indicative of cell damage. The details for all these methods are described in Supplementary materials (Supplementary Texts 5–7). Each experiment was performed at least in triplicate.

#### RNA Extraction and Genome-Wide RNA Sequencing for Transcriptomic Analysis

To study how herbicide exposure might affect the bacterial gene expression, we conducted transcriptomic analysis of *E. coli* DH5α strain in the absence and presence of all herbicides (at concentration of 10 mg/l) after 6 h exposure at 30°C. Total RNA was extracted from the strains using the QIAGEN miRNeasy Mini Kit (Cat #217184, Valencia, CA, USA) manufacturer’s protocol. RNA quality and concentration were measured using Qubit RNA Assay Kit in a Qubit 3.0 Fluorometer (Life Technologies). Furthermore, RNA integrity was assessed using the RNA Nano 6000 Assay kit by Agilent Bioanalyzer 2100 (Agilent Technologies). A 1 μg high-quality RNA (RNA integrity number >7) was used as input material for the library preparation. Ribosomal RNA was removed using Epicentre Ribo ZeroTM rRNA Removal Kits (Epicentre, USA) and sequencing libraries were subsequently prepared using Library Prep Kit for Illumina (Vazyme, catalogue no. NR603) following the manufacturer’s protocols. RNA samples were then submitted to Guangdong Magigene Biotechnology Co. Ltd for strand-specific cDNA library construction and paired-end sequencing (HiSeq platform, Illumina, USA). Bioinformatics for whole-genome RNA sequencing were performed following a previous study (Wang et al. 2019). Briefly, clean reads were obtained by removing low-quality reads, reads containing adaptor and reads with more than 10% of unidentified base information. The individual libraries were converted into the FASTQ format and gene expression was calculated as fragments per kilobase of a gene per million mapped reads (FPKM). Differences in fold changes between different groups were calculated by log 2 fold-change between control and herbicide-treated samples using the DESeq2 package (Love et al. 2014). The differentially expressed genes between the two groups were identified by considering both fold changes (|log2FC| > 1) and adjusted *P*-values (false-discovery rate corrected *P *<* *0.05) (Li et al. 2019).

### Collection and Analysis of Agricultural Field Data

#### Field Sites and Soil Sampling

To test the effect of herbicide on ARG and MGE abundances in agricultural fields, 21 agricultural soil sites across 11 provinces were selected for sampling (from 22.78°N to 48.89°N and 100.97°E to 130.40°E across eastern China, Supplementary fig. S22). Based on the oral communication with farmers, glyphosate and glufosinate were the main herbicides used in the field sampling sites. As glyphosate had the relatively largest effect in our lab experiments, we focused on quantifying only glyphosate concentrations in the field samples.At each location, two sites were sampled: herbicide-exposed field sites that had consecutively been under glyphosate use at least 10 years with recommended application rate of 1.455–2.895 kg/ha (herbicides were typically applied for 2–4 times at each field site based on the cultivated crop), and correspondingly, 21 control sites with no exposure to any herbicides during the past 5 years (Supplementary table S9). Each of the paired sites were located in adjacent fields with less than 1 km distance in between. At each site, three 50 m^2^ plots were sampled using six soil cores (500 g per core) at a depth of 0–10 cm per plot in June 2020. All soil samples per site were mixed and homogenized manually before the analyses resulting in total of 42 soil samples. qPCR analysis of target ARGs and MGEs were performed as described previously (Liao et al. 2018).

#### Measuring Glyphosate Residues in Soil Samples Using LC-MS/MS

The glyphosate residues in the collected soil samples soils were analyzed using liquid chromatography-tandem mass spectrometry as described previously (Silva et al. 2018). Glyphosate concentrations were determined from soil washes in aliquots through High-performance liquid chromatography-tandem mass spectrometry (HPLC-MS)/MS following the same extraction and derivatization methods, chemicals, mobile phases, column characteristics and instrumentation conditions as described in Silva et al (2018). Further details and methods of these analyses are described in Supplementary Text 8.

#### Statistical Analysis

Bacterial total abundances, community diversity and richness were estimated using 16S rRNA gene copies, alpha diversity (Shannon and observed ASV) and beta diversity (Weighted-UniFrac distance) using the q2-diversity pipeline within QIIME2. Bacterial community and resistome structure were visualized using principal co-ordinates analysis (PCoA, Weighted-UniFrac distance) and nonmetric multidimensional scaling (NMDS, Bray–Curtis distance) ordination, respectively. PERMANOVA (Adonis test) was used to determine the significance of different treatment on the soil microbiome as well as antibiotic resistome. The overall mean differences between treatments were analyzed using repeated measures (with time structured data) or ANOVA (one-way or two-way) followed by multiple comparison based on Tukey HSD test. For nonparametric tests, such as Wilcoxon signed-rank and Adonis tests, statistical significance was determined based on 999 permutations. In all analyses, *P*-values smaller than 0.05 were used to indicate a statistically significant difference. All statistical analyses were performed using the SPSS 19 (IBM, USA) and R 3.5.1 (Team RC^2019^) using vegan (v2.5-6), ggplot2 (3.3.1), tidyverse (3.0.1), ggrepel (0.8.2), and pheatmap (1.0.12) packages.

## Data Availability

Sequencing data generated from both amplicon and shotgun metagenomes in this study have been deposited with the NCBI SRA (accession no: PRJNA634745) and are publicly available. The authors declare that the other main data supporting the findings of this study are available within this article and in the Supplementary information files. Additional data supporting the findings of this study are available from the corresponding author upon reasonable request.

## Code Availability

All code used in this study are available from the corresponding author upon request.

## Supplementary Material

Supplementary data are available at *Molecular Biology and Evolution* online.

## Supplementary Material

msab029_Supplementary_Data
