# Supplementary material for: Herbicide Selection Promotes Antibiotic Resistance in Soil Microbiomes
Source: Mol Biol Evol. 2021 Feb 16;38(6):2337–50. doi: 10.1093/molbev/msab029 (PMC8136491; doi:10.1093/molbev/msab029)
Supplement: msab029_Supplementary_Data [file msab029_supplementary_data.zip › SupplementaryMethodsAndFigures.pdf]

Supplementary Information

Herbicide selection promotes antibiotic resistance in soil microbiomes

Hanpeng Liao[1], Xi Li[1], Qiue Yang[1], Yudan Bai[1], Peng Cui[1], Chang Wen[1], Chen Liu[1], Zhi Chen[1], Jiahuan Tang[1], Jiangang Che[1], Zhen Yu[2], Stefan Geisen[3], Shungui Zhou[1#], Ville-Petri Friman[4#], Yong-Guan Zhu[5]

Affiliations

[1] Fujian Provincial Key Laboratory of Soil Environmental Health and Regulation, College of Resources and Environment, Fujian Agriculture and Forestry University, Fuzhou 350002, China.

[2] Guangdong Key Laboratory of Integrated Agro-environmental Pollution Control and Management, Guangdong Institute of Eco-environmental Science & Technology, Guangzhou 510650, China.

[3] Laboratory of Nematology, Wageningen University, 6700AA Wageningen, Netherlands.

[4] Department of Biology, University of York, Wentworth Way, YO10 5DD, York, UK.

[5] Key Lab of Urban Environment and Health, Institute of Urban Environment, Chinese Academy of Sciences, Xiamen 361021, China.

[#]Corresponding authors

Correspondence to Shun-Gui Zhou (sgzhou@soil.gd.cn) and Ville-Petri Friman (ville.friman@york.ac.uk)

Supplementary methods

Supplementary Text 1: Determination of residual herbicides by HPLC in soil microcosm experiments

1.1 Determination of glyphosate and glufosinate residues at the end of soil microcosm experiment

The remaining glyphosate and glufosinate residues in soil were measured using high-performance liquid chromatography (HPLC) with pre-column derivatization of 9-fluorenylmethyl chloroformate (FMOC) as described previously(Druart, et al. 2011). The HPLC system (U3000, ThermoFisher Scientific, Bremen, Germany) consisted of auto sampler and fluorescence detector. Following FMOC derivatization was used: a 2.47 ml of extract and 30 μL of internal standard were derivatized with 250 μL of FMOC and 250 μL of borate buffer under agitation (by magnetic stirrer) for 1 h at room temperature. In order to eliminate the excess of FMOC, a liquid-liquid extraction with 2 ml of ethyl ether was performed. The separation was performed using a reverse phase C18 column (300 × 3.9 mm, i.d.), with H_3PO_4 at 0.2% (solvent A) / acetonitrile (solvent B) eluent as the mobile phase at a flow rate of 1 ml/min. The column oven temperature was set to 25 °C and 20 μL injection volume was used. The gradient was discontinuously increased from 10% of solvent B to 45% in 32 min and then decreased back to 10% in 3 min. Detection of FMOC derivatives of each herbicide was done at λ_{ex} = 260 nm and λ_{em} = 310 nm.

1.2 Determination of dicamba concentration

Concentration of dicamba was measured using a method described previously (Shin, et al. 2011) based on a HPLC system coupled with UV detector. The C18 column was used to separate dicamba. The mobile phase consisted of 0.02% phosphoric acid aqueous solution (pH 2.5)-ACN (60:40, v/v), the flow rate was set to 0.8 mL/min in isocratic mode and the analytes were detected at a wavelength of 230 nm. The column oven temperature was set to 25 °C and 10 μL injection volume was used.

Supplementary Text 2: Metagenomic sequencing and data analysis

Briefly, approximately 1 μg DNA was used to construct library with a 300 bp insert size, followed by sequencing using Illumina HiSeq X ten platform (Guangdong Magigene Biotechnology Co. Ltd, Guangzhou, China, 2×150 paired reads). The raw the sequences with average quality scores < 30 (Q30) or length < 50 bp were removed using Trimmomatic, resulting in 75.1 million clean reads with average of 6.2 Gb clean reads for each sample. More detailed information about the dataset and individual samples are summarized in Supplementary Table S8. Resistome and mobilome were analysed using local ARGs-OAP (v2.2) based on the clean reads (Yin, et al. 2018). The ARG reads were identified against the SARG database at the cut-off of 10^{-7} E-value, 80% identity and 75% hit length. SARG database contains 24 ARG types, 1208 ARG subtypes, and 12307 non-redundant reference sequences (Yin, et al. 2018). Resistance 'types' represent the class of antibiotics to which ARGs confer resistance, while 'subtypes' represent individual kind of ARGs, such as subtype *tetA* of tetracycline resistance genes. The reads were analysed against a database of 30 essential single-copy gene families, which are found in nearly all bacteria and archaea, and can be considered as bacterial and archaeal PhyEco marker genes (Nayfach and Pollard 2015). The average coverage of essential single-copy gene was used to calculate the cell numbers in each sample. To compare the ARGs between samples, we normalized ARG abundances with numbers of cells in each sample based on essential single-copy gene numbers. The relative ARG abundances were then analysed and presented as ARG copies per cell (copies/cell). Quantification and analysis of mobilome (all MGEs) was analysed similarly to ARGs using a MGE-specific reference database(Parnanen, et al. 2018).

Supplementary Text 3: Real-time quantitative PCR to determining ARG and MGE gene abundances

The primers, annealing temperatures, and amplification protocols for all gene targets are listed in the supplementary material (Table S6). The qPCR and plasmid constructions were conducted according to a previous protocol (Liao, et al. 2018) by using the LightCycler 96 System (Roche, Mannheim, Germany). Briefly, the plasmids carrying target genes were obtained from TA clones and extracted by using a TIANGEN pure Mini Plasmid kit (Tiangen, Beijing, China). The standard plasmid concentrations (ng/mL) were determined with the Nanodrop ND-2000 (Thermo Fisher Scientific, Wilmington, USA) to calculate gene copy concentrations (copies/mL). The qPCR was carried out in 96-well plates containing 10 μL of GoTaq qPCR Master Mix (Promega, Madison, USA), 1.5 μL each of forward and reverse primers (4 mmol/L), 1 μL of template genomic DNA and 6 μL of nuclease-free water. Each qPCR run began with 2 min of initial denaturation at 95 °C, followed by 40 cycles of denaturation at 95 °C for 30 s, annealing for 30 or 45 s according to the length of target at the primer-specific annealing temperature, and extension for 30 s at 72 °C. The amplification efficiencies of different PCR reactions ranged from 90% to 110% with R^2 values higher than 0.99 for all standard curves. Each reaction was run in triplicate along with standard curves and a negative control where the template genomic DNA was replaced with DNA-free water.

Supplementary Text 4: Detection of RP4 plasmid genes in recipient cells using PCR

Conjugation of plasmid RP4 to recipient cells was verified using PCR targeting global regulatory genes (*korA*, *korB*, and *trbA*), conjugation-related genes (*trbBp*, *trfAp*, *traF*, and t*raJ*), outer membrane protein-encoding genes (*ompA*, *ompF*, and *ompC*), and an oxidative stress regulatory gene (*rpoS*) using PCR. The primers for all gene targets are listed in the supplementary material (Supplementary Table S10). The PCR mixtures consisted of 10 μL of 2 × Premix Ex Taq (TaKaRa, Dalian, China), 0.4 μL of each primer (10 μM final concentrations), 1 μL of DNA template, and 8.6 μL of distilled H_2O.

Supplementary Text 5: Measurement of cell membrane permeability

The hydrolysis rate of the o-nitrophenyl-β-D-galactopyranoside (ONPG) by bacterial cells was measured to quantify cell membrane permeability caused by herbicide exposure using previously described methods(Jin, et al. 2020). The donor and recipient strains were incubated at 30 °C overnight in LB medium and shaken at 150 rpm. Bacterial cells were then collected by centrifugation at 6000×g for 5 min at 4 °C, washed five times with phosphate-buffered saline (PBS) (1 × PBS, pH = 7.2) and resuspended to PBS in cell densities of 1×10^8 CFU/mL. The cells with an approximate concentration of 10^{7-8} cfu/mL were exposed to herbicides (10 mg/L) for 6 h at 30 °C. Herbicide-treated *E. coli* was dispensed into a sterile 1.5-mL tube containing 0.1 mL of 5 mmol/L ONPG (Sigma-Aldrich) as the substrate. The mixture was let to react in a water bath at 37 °C for 2 hours, after 6 μL of 0.2 mol/L Na_2CO_3 solution was added to terminate the reaction between the enzyme and substrate. Absorbance values were determined at 420 nm using a microplate reader (Infinite® 200 PRO, Tecan, Swiss). As a control, similar measurements were conducted for *E. coli* suspensions grown in the absence of herbicides. Each experiment was performed at least in triplicate.

Supplementary Text 6: Reactive oxygen species (ROS) measurement

To determine the ROS levels in bacteria, cells were analysed by DCF-DA/H_2DCFDA-cellular ROS detection assay kit (Abcam, USA) following manufacturer's instructions. Briefly, the bacterial suspensions, herbicide-treated or not, were adjusted to 10^6 cfu/mL in 0.85% (w/v) saline in a 1.5-mL tube. Subsequently, 20 μmol/L DCF-DA was added to the suspension and then immediately pre-incubated and protected from light at 37 °C for 45 min. After incubation, the suspension was transferred to a 96-well plate containing different herbicides at 0 (no-herbicide control) and 10 mg/L concentration at 37 °C for 4 h. To exclude the background fluorescence of the bacteria, a blank control (with bacteria without any herbicides) was used.

All samples were measured at an excitation wavelength of 485 nm and an emission wavelength of 535 nm indicative of ROS activity. Each experiment was performed at least in triplicate.

Supplementary Text 7: Detection of bacterial antioxidant enzyme activity

Herbicide-treated bacteria at a concentration of 10^8 cfu/mL were prepared according to Supplementary Text 5, to assay the oxidative stress-response as antioxidant enzyme activity. After sonication at 20 kHz (150 W) for 10 min (VCX750, Sonics, USA), the collected samples were centrifuged at 5,000 × *g*, 4 °C for 3 min. The levels of superoxide dismutase (SOD) and catalase (CAT) in the supernatant were assessed using appropriate kits obtained from the Nanjing Jiancheng Bioengineering Institute (Nanjing, China) following manufacturers' instructions. The absorbance values of each indicator kit were determined at 405 (CAT) and 550 nm (SOD), respectively, using a plate reader (Infinite 200 PRO®, Tecan, Swiss). As controls, 10^7 and 10^8 cfu/mL bacterial suspensions grown in the absence of herbicides were used. Each experiment was performed at least in triplicate.

Supplementary Text 8: Determination of glyphosate residues in field samples using HPLC-MS/MS

The samples were analyzed using a Waters Acquity™ UPLC® coupled with an Acquity™ TQD tandem mass spectrometer (Waters Co., Milford, MA). An Acquity™ UPLC BEH-Amide (1.7 µm, 2.1 × 100 mm) column was utilized to achieve the separation and retention of analytes. The column was injected with a sample volume of 5.0 µL with a flow rate of 0.300 mL/min, a mobile phase isocratic at 30% water/70% acetonitrile with a run time of 5 minutes at 25°C. The detection and quantification of glyphosate were performed in negative ESI-MS mode. Briefly, glyphosate analytes were identified according to the retention time and peak shape of isotopically-labelled internal standards, glyphosate (1,2-^{13}C,^{15}N). The responses of the analytes were normalized according to the response of the isotopically-labelled internal standards. A

calibration curve was established from 0.01 to 2 μg/mL with a R^2 value of 0.999. All calibration curves presented satisfactory linearity of response versus concentration, with correlation coefficients ≥0.99 and individual residuals within ±25%. Similar recovery values (75-110%) were observed in soil samples fortified with the same glyphosate standard. The concentration of glyphosate was measured independently for two aliquots (replicates) per collected sample. The limit of detection and quantification of glyphosate was set to 0.02 mg/kg and 0.03 mg/kg, respectively. All the validation parameters and quality control criteria used were in line with those described in the guidance document for pesticides residues analysis in food and feed (REPORT 2005).

Supplementary results

Herbicides significantly increased the abundances of genes related to antibiotic resistance and mobile elements

As a separate validation to metagenomic sequencing, quantitative PCR was used to confirm that the relative abundance of 27 ARGs (including tetracycline, sulfonamide, aminoglycoside and macrolide resistance genes) and five MGEs (two integrases (*intI1*, *intI2*), two plasmids (*ISCR1*, *IncQ*), one transposon (*Tn916*)) relative to control treatment at 0d, 30d and 60d sampling time points. Both herbicide exposure treatment ($F_{3,8}$=6.827, p=0.013) and exposure time ($F_{2,7}$=2380.795, p<0.001) significantly increased the ARG and MGE abundances during the experiment (Extended Data Fig. 10a-b). Total ARG abundances were higher relative to the control treatment in all herbicide treatments at 30d ($F_{3,8}$=41.642, p<0.001) and 60d sampling time points ($F_{3,8}$=14.956, p=0.0012). Specifically, dicamba exposure increased the abundance of tetracycline and sulfonamide resistance genes (Extended Data Fig. 10c, $F_{3,8}$=55.191, p<0.001), while glufosinate and glyphosate increased aminoglycoside resistance genes (Extended Data Fig. 10c, $F_{3,8}$=28.206, p<0.001). A similar increase in MGE abundances was observed under herbicide exposure

relative to control treatment and MGE abundances increased in time throughout the experiment (Extended Data Fig. 10b, 30d: $F_{3,8}$=14.948, p=0.001; 60d: $F_{3,8}$=17.69, p<0.001) reaching 1.62-fold, 2.04-fold and 1.47-fold increases in glyphosate, glufosinate and dicamba treatments, respectively (at 60d sampling time point). Two plasmid genes (*ISCR1* and *IncQ*) became the most prevalent MGEs ($F_{3,8}$=28.206, p<0.001) by the 60d sampling time point (Extended Data Fig. 11). Moreover, a strong linear correlation between ARG and MGE abundances was observed (R^2 =0.759, $p < 0.0001$, Extended Data Fig. 12a), specifically, between ARGs and both *IncQ* (R^2 =0.608, $p = 0.0017$, Extended Data Fig. 12b) and *ISCR1* (R^2 =0.903, $p < 0.0001$, Extended Data Fig. 12c) plasmid genes.

To confirm the enrichment of antibiotic resistant bacteria under herbicide treatment, we used cultivation-based methods to isolate antibiotic resistant bacteria (ARB) at 30d sampling point using amoxicillin, erythromycin, chloramphenicol and tetracycline containing selective agar plates. Herbicide exposure significantly increased the total abundance of culturable ARBs compared to control group ($F_{3,8}$=201.424, $p < 0.001$). Specifically, the total number of ARBs under glufosinate treatment was 1.8-fold higher than the control treatment (change from $6.2 \pm 0.27 \times 10^3$ to $11.3 \pm 0.068 \times 10^3$ CFU/g). Furthermore, changes in the abundance of ARBs depended on the specific resistance genes and exposure to certain type of herbicide. For example, Glyphosate exposure significantly increased the number of erythromycin ($F_{3,8}$=18.204, $p < 0.001$) and amoxicillin ($F_{3,8}$=125.429, p<0.001) resistant bacteria (Extended Data Fig. 13a-c), while chloramphenicol-resistant bacteria increased especially in glufosinate and dicamba herbicide treatments. In contrast, no significant differences for tetracycline resistant bacteria were observed in any of the herbicide treatments (Extended Data Fig. 13d).

Supplementary references used in Table 1

1 Favrot, L., Blanchard, J. S. & Vergnolle, O. Bacterial GCN5-Related N-Acetyltransferases: From Resistance to Regulation. Biochemistry-us 55, 989-1002, doi:10.1021/acs.biochem.5b01269 (2016).

2 Tanaka, Y., Iwaki, S. & Tsukazaki, T. Crystal Structure of a Plant Multidrug and Toxic Compound Extrusion Family Protein. Structure 25, 1455-1460.e1452 (2017).

3 Zheng, T. & Nolan, E. M. Enterobactin-Mediated Delivery of β-Lactam Antibiotics Enhances Antibacterial Activity against Pathogenic Escherichia coli. J Am Chem Soc 136, 9677-9691, doi:10.1021/ja503911p (2014).

4 May, K. L. & Grabowicz, M. The bacterial outer membrane is an evolving antibiotic barrier. Proc Natl Acad Sci USA 115, 8852-8854 (2018).

5 Locher, K. P. Mechanistic diversity in ATP-binding cassette (ABC) transporters. Nat Struct Mol Biol 23, 487-493 (2016).

6 Ghai, I. & Ghai, S. Understanding antibiotic resistance via outer membrane permeability. Infect Drug Resist 11, 523-530, doi:10.2147/IDR.S156995 (2018).

7 Gupta, K., Liao, J., Petrova, O. E., Cherny, K. E. & Sauer, K. Elevated levels of the second messenger c-di-GMP contribute to antimicrobial resistance of Pseudomonas aeruginosa. Mol Microbiol 92, 488-506 (2014).

8 Wells, T. J., Tree, J. J., Ulett, G. C. & Schembri, M. A. Autotransporter proteins: novel targets at the bacterial cell surface. Fems Microbiol Lett 274, 163-172, doi:10.1111/j.1574-6968.2007.00833.x (2007).

9 Avalos Vizcarra, I. et al. How type 1 fimbriae help Escherichia coli to evade extracellular antibiotics. Sci Rep 6, 18109 (2016).

10 Ibacache-Quiroga, C., Oliveros, J. C., Couce, A. & Blázquez, J. Parallel evolution of high-level aminoglycoside resistance in escherichia coli under low and high mutation supply rates. Front Microbiol 9, doi:10.3389/fmicb.2018.00427 (2018).

11 Fischer, E., Wolf, H., Hantke, K. & Parmeggiani, A. Elongation factor Tu resistant to kirromycin in an Escherichia coli mutant altered in both tuf genes. Proc Natl Acad Sci USA 74, 4341-4345, doi:10.1073/pnas.74.10.4341 (1977).

12 Carr, J. F., Hamburg, D.-M., Gregory, S. T., Limbach, P. A. & Dahlberg, A. E. Effects of Streptomycin Resistance Mutations on Posttranslational Modification of Ribosomal Protein S12. J Bacteriol 188, 2020-2023, doi:10.1128/jb.188.5.2020-2023.2006 (2006).

13 Wang, C., Lin, X., Li, L. & Lin, S. Differential Growth Responses of Marine Phytoplankton to Herbicide Glyphosate. PloS one 11, e0151633-e0151633, doi:10.1371/journal.pone.0151633 (2016).

14 Cummins, I. et al. Key role for a glutathione transferase in multiple-herbicide resistance in grass weeds. Proc Natl Acad Sci USA 110, 5812-5817 (2013).

15 Christoffoleti, P. J., Figueiredo, M. R. A. d., Peres, L. E. P., Nissen, S. & Gaines, T. Auxinic herbicides, mechanisms of action, and weed resistance: A look into recent plant science advances Scientia Agricola 72, 356-362 (2015).

16 Abell, L. M. Biochemical Approaches to Herbicide Discovery: Advances in Enzyme Target Identification and Inhibitor Design. Weed Sci 44, 734-742 (1996).

17 Otoshi, A., Yoshimura, K., Miyagawa, Y. & Shigeoka, S. Isolation of genes involved in stress tolerance by activation tagging Science Access 3 (2001).

18 Dahabiyeh, L. A., Bustanji, Y. & Taha, M. O. The herbicide quinclorac as potent lipase inhibitor: Discovery via virtual screening and in vitro/in vivo validation. Chemical biology & drug design 93, 787-797, doi:10.1111/cbdd.13463 (2019).

19 Délye, C., Duhoux, A., Pernin, F., Riggins, C. W. & Tranel, P. J. Molecular Mechanisms of Herbicide Resistance. Weed Sci 63, 91-115, 125 (2015).
20 Ohta, D., Mori, I. & Ward, E. Inhibitors of imidazoleglycerolphosphate dehydratase as herbicides. Weed Sci 45, 610-620 (1997).

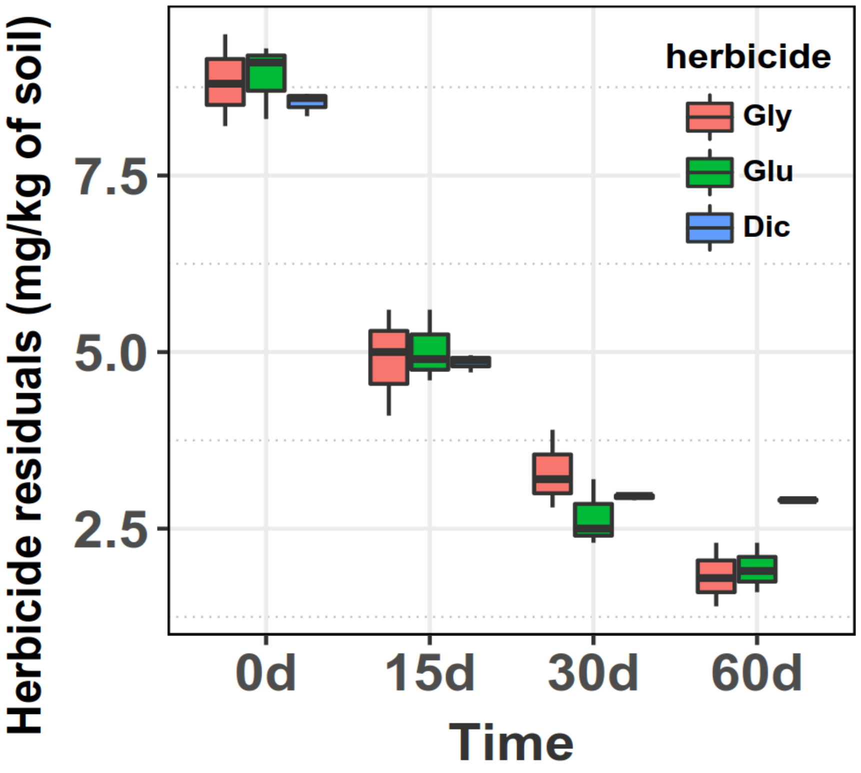

Figure 1. Dynamics of herbicide degradation during the soil microcosm experiment after 15, 30 and 60 days of initial application of glyphosate (Gly), glufosinate (Glu) and dicamba (Dic). Box plots encompass the 25–75th percentiles, the whiskers show the minimum and maximum values and the midline indicates the median (n=3 biological replicates).

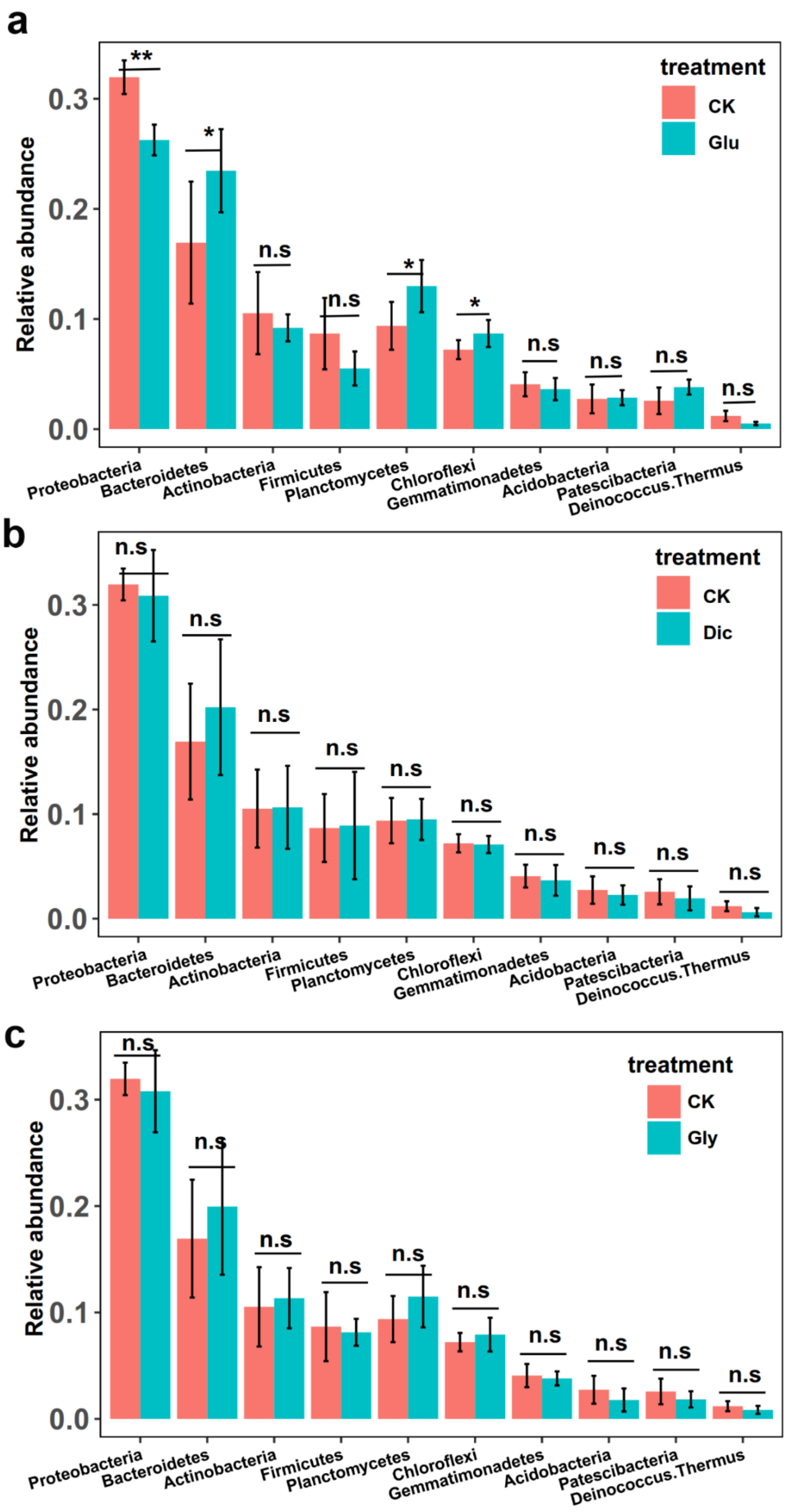

Figure 2. Changes in the relative bacterial phyla abundances between glufosinate (a), dicamba (b) and glyphosate (c) treatments relative to no-herbicide control treatment (CK) averaged over 30d and 60d sampling timepoints. Data show mean ± SD of three biological replicates (n=3 per treatment) and significances are shown as * p<0.05, **p <0.01, while n.s indicates p > 0.05 based on paired Student's t-test.

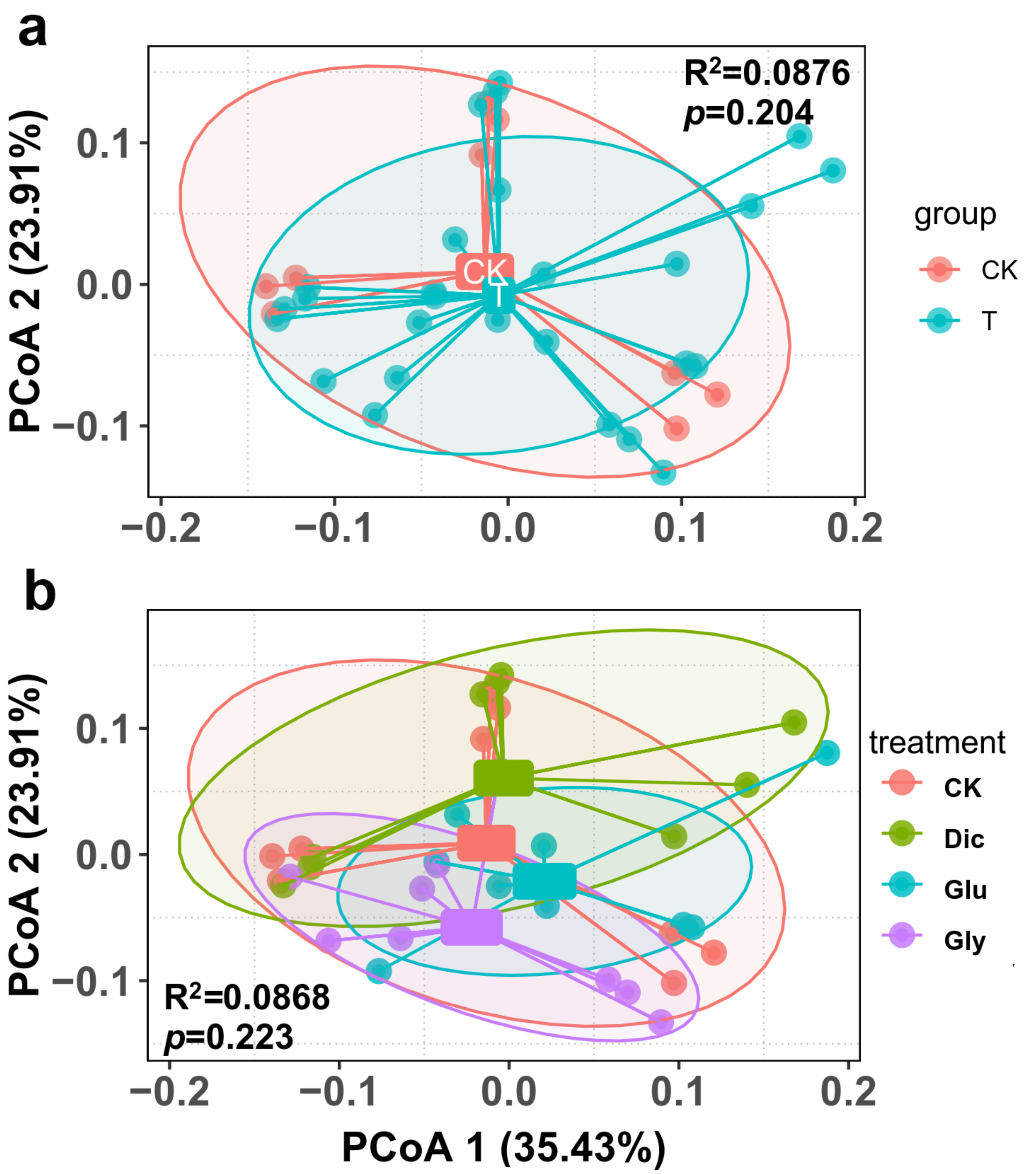

Figure 3. Comparison of bacterial community composition based on distance-based (weighted Unifrac) PCoA analysis between herbicides treatment and control treatments (n=9 biological replicates per treatment) during the soil microcosm experiment. (a) Shows the mean comparison of the bacterial communities between herbicide-exposed (T) and no-herbicide control treatment (CK). (b) Shows the comparison of the bacterial communities between glyphosate (Gly), glufosinate (Glu), dicamba (Dic) and no-herbicide (CK) treatments. In both panels, data is based on 16S rRNA amplicons and averaged over 15d, 30d and 60d sampling time points. Adonis test was used to identify significant differences between the treatments.

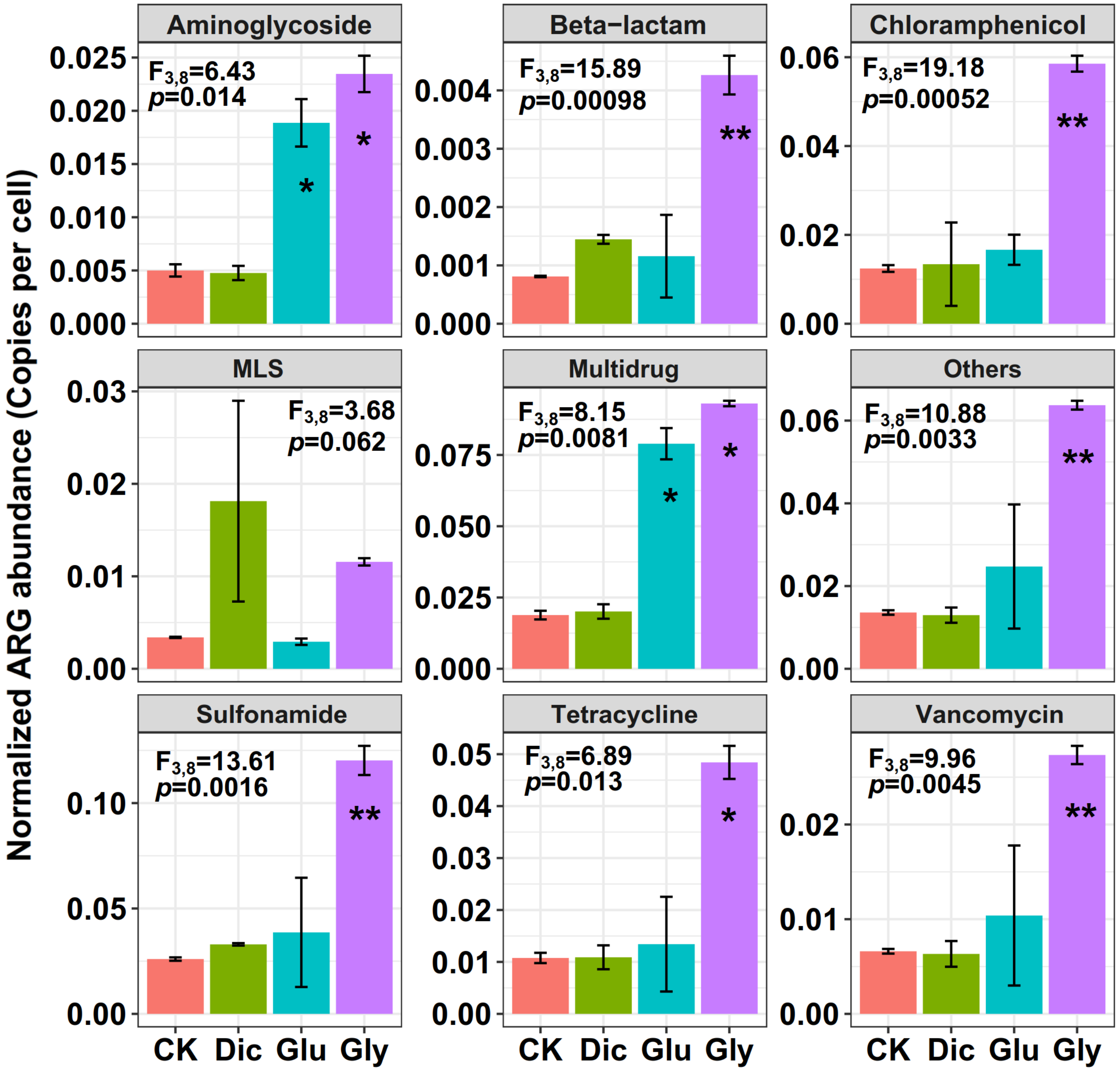

Figure 4. The effect of herbicide exposure on ARG type abundances in control (CK), dicamba (Dic), glufosinate (Glu) and glyphosate (Gly) treatments based on metagenomic analysis at 30d sampling time point. Asterisk denotes significant differences (* $p<0.05$, ** $p<0.01$) compared to no-herbicide treatment based on Bonferroni-adjusted Tukey test. Data show mean ± SD of three biological replicates (n=3 per treatment).

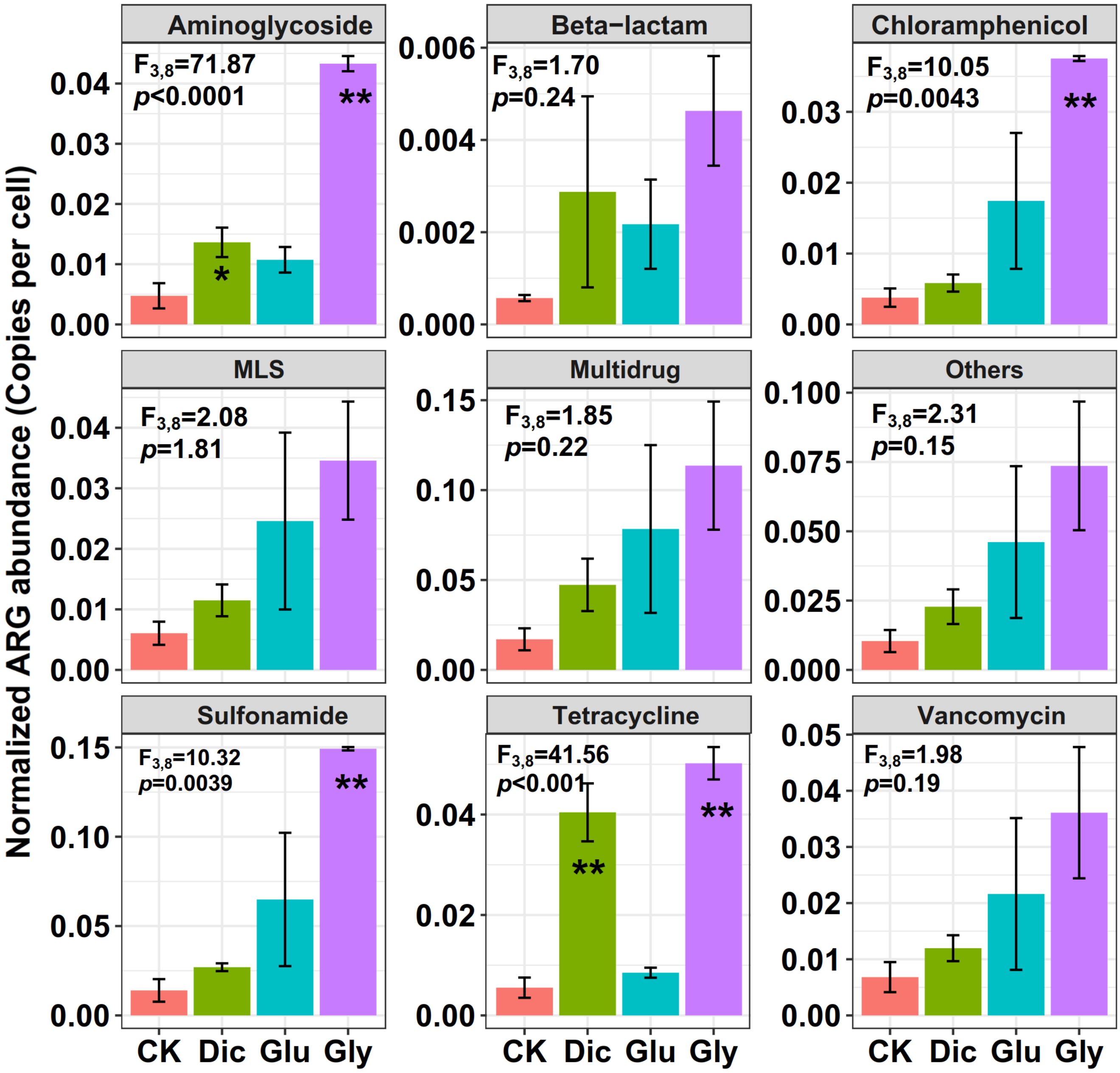

Figure 5. The effect of herbicide exposure on ARG type abundances in control (CK), dicamba (Dic), glufosinate (Glu) and glyphosate (Gly) treatments based on metagenomic analysis at 60d sampling time point. Asterisk denotes significant differences (* $p<0.05$, ** $p<0.01$) compared to no-herbicide treatment based on Bonferroni-adjusted Tukey test. Data show mean ± SD of three biological replicates (n=3 per treatment).

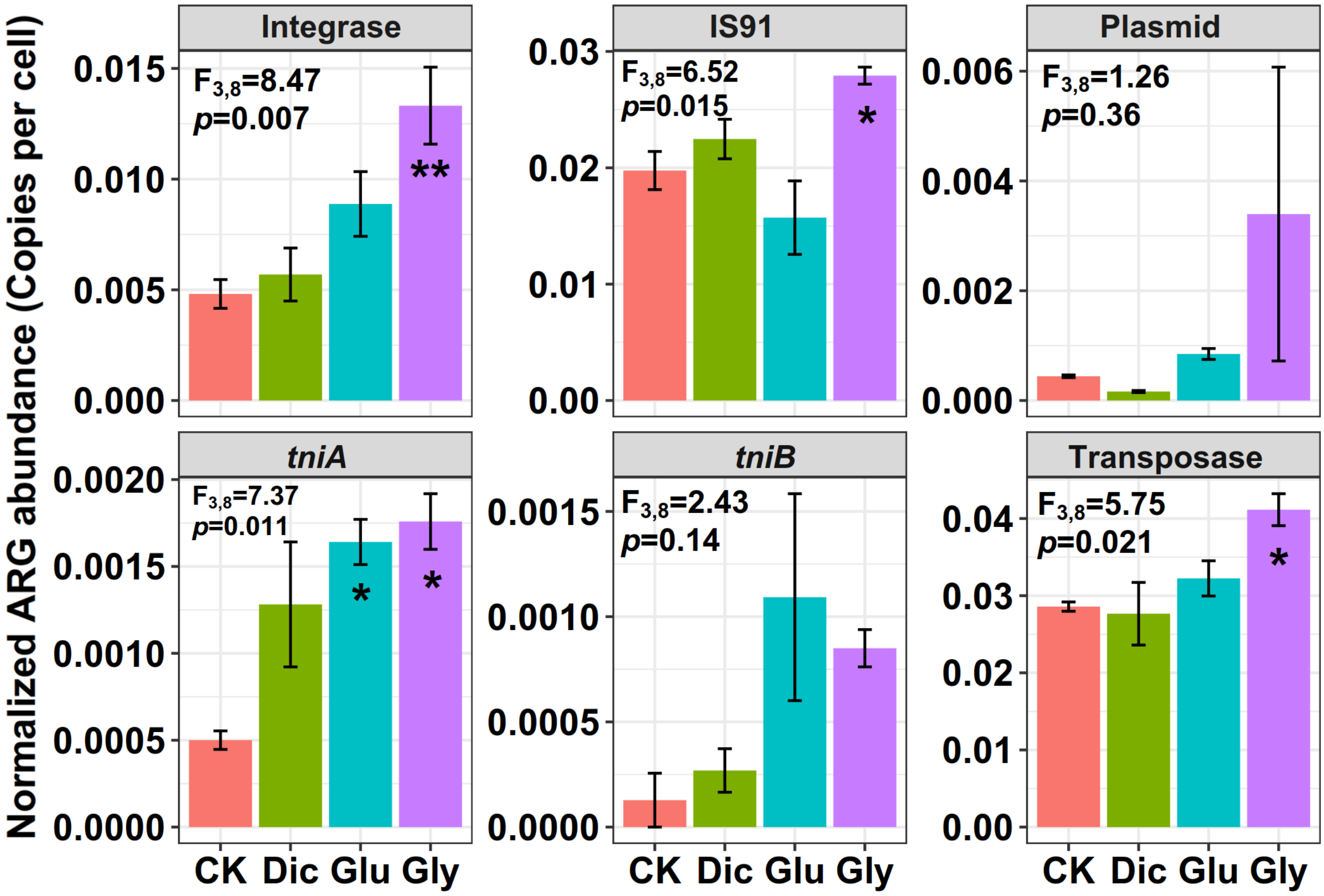

Figure 6. The effect of herbicide exposure on MGE type abundances in control (CK), dicamba (Dic), glufosinate (Glu) and glyphosate (Gly) treatments based on metagenomic analysis at 30d sampling time point. Significant differences (* p<0.05, ** p <0.01) compared to no-herbicide treatment based on Bonferroni-adjusted Tukey test. Data show mean ± SD of three biological replicates (n=3 per treatment).

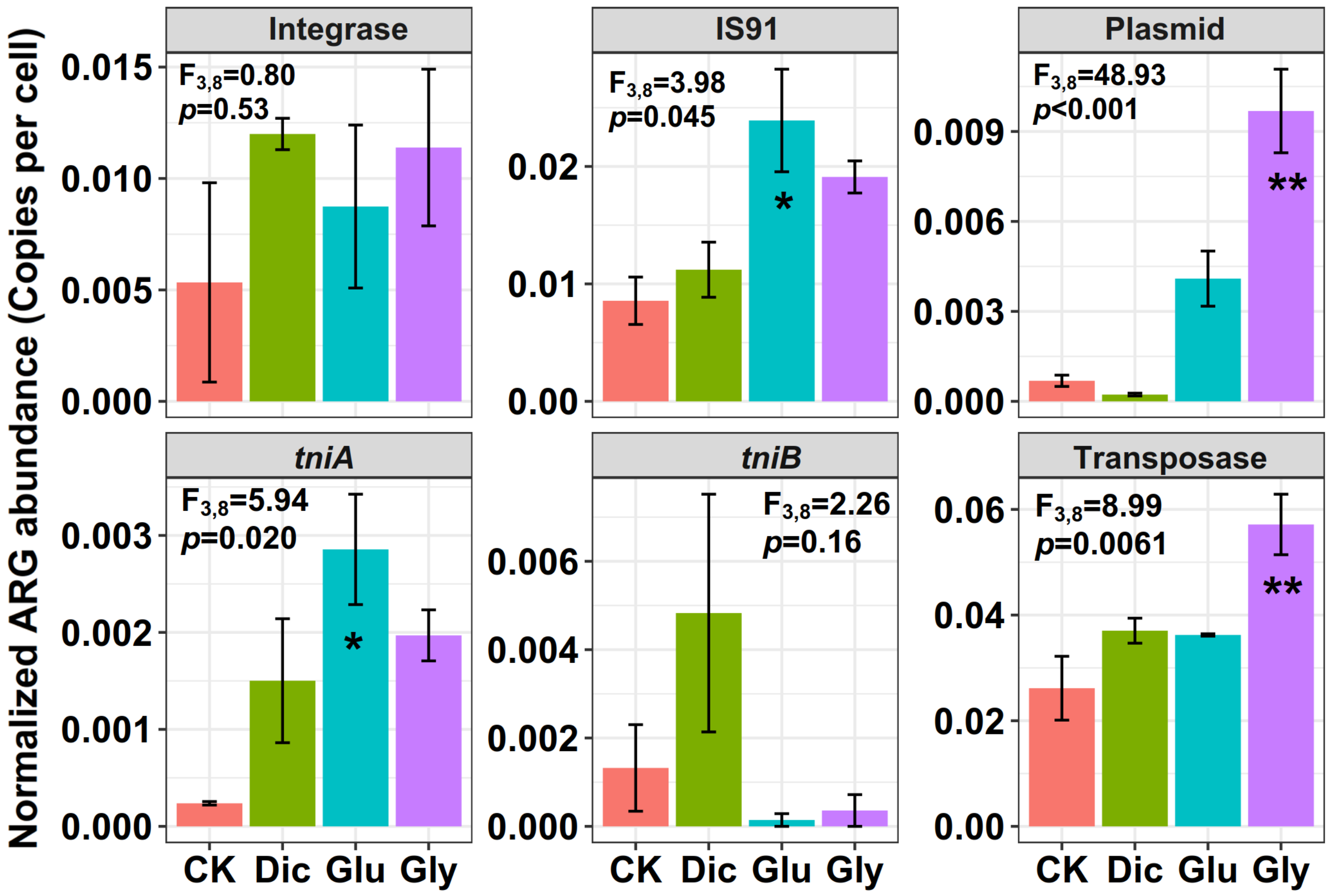

Figure 7. The effect of herbicide exposure on ARG type abundances in control (CK), dicamba (Dic), glufosinate (Glu) and glyphosate (Gly) treatments based on metagenomic analysis at 60d sampling time point. Asterisk denotes significant differences (* $p<0.05$, ** $p<0.01$) compared to no-herbicide treatment based on Bonferroni-adjusted Tukey test. Data show mean ± SD of three biological replicates (n=3 per treatment).

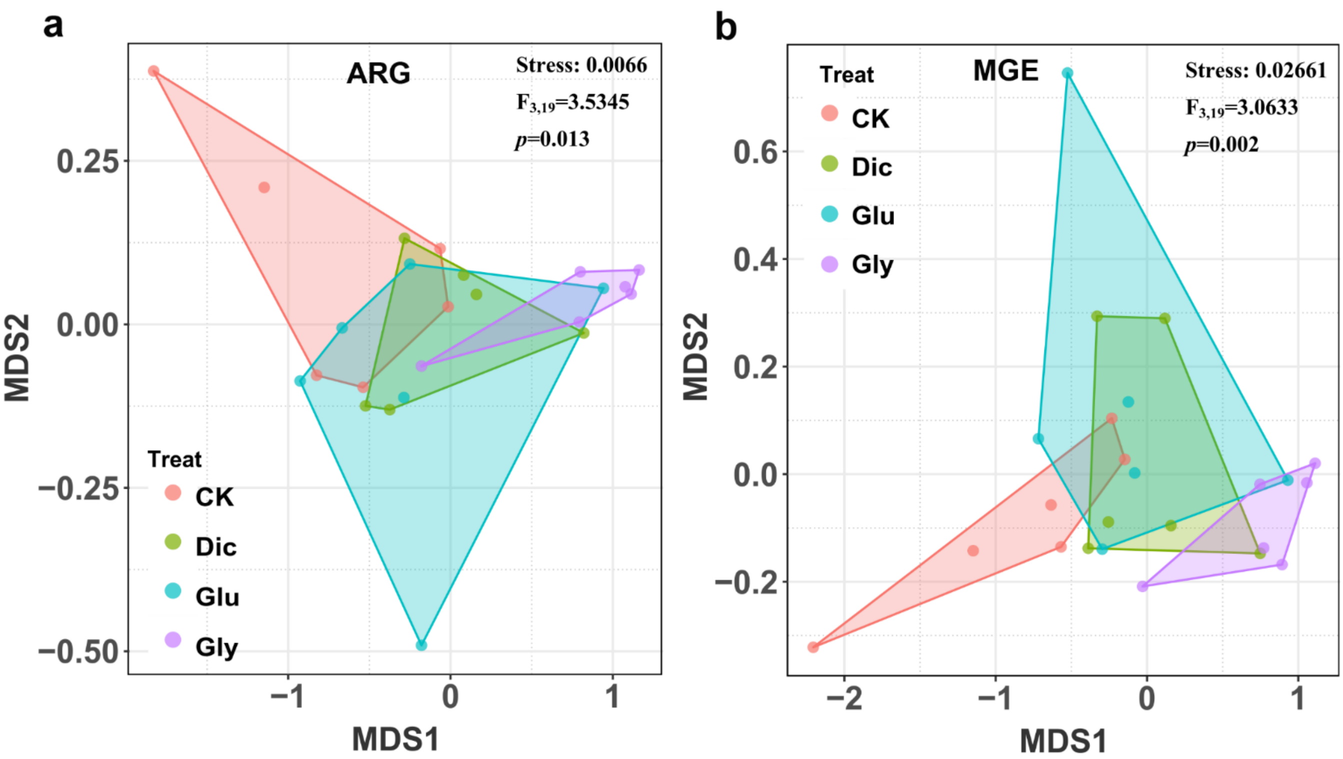

Figure 8. Changes in the structure of soil resistome (a) and mobilome (b) in control (CK), dicamba (Dic), glufosinate (Glu) and glyphosate (Gly) treatments based on Two-dimensional NMDS analysis (Bray-Curtis dissimilarity) averaged over 30d and 60d sampling time points. Differences between treatments were assessed using PERMANOVA (Adonis test) and all ARG and MGE abundances were normalized against the number of bacterial cells. Data show mean ± SD of three biological replicates (n=3 per treatment).

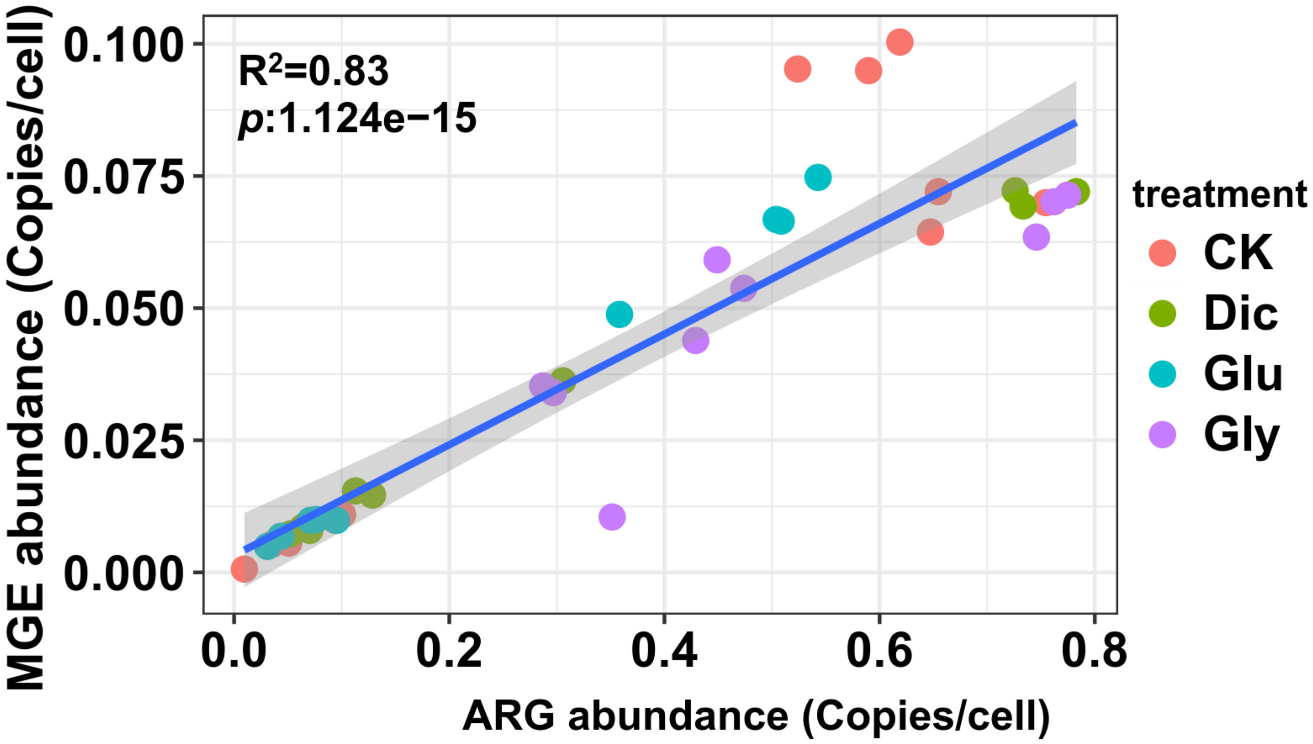

Figure 9. Positive relationship between the relative MGE and ARG abundances over control (CK), dicamba (Dic), glufosinate (Glu) and glyphosate (Gly) treatments (linear regression analysis based on metagenomics data, n=3 per treatment).

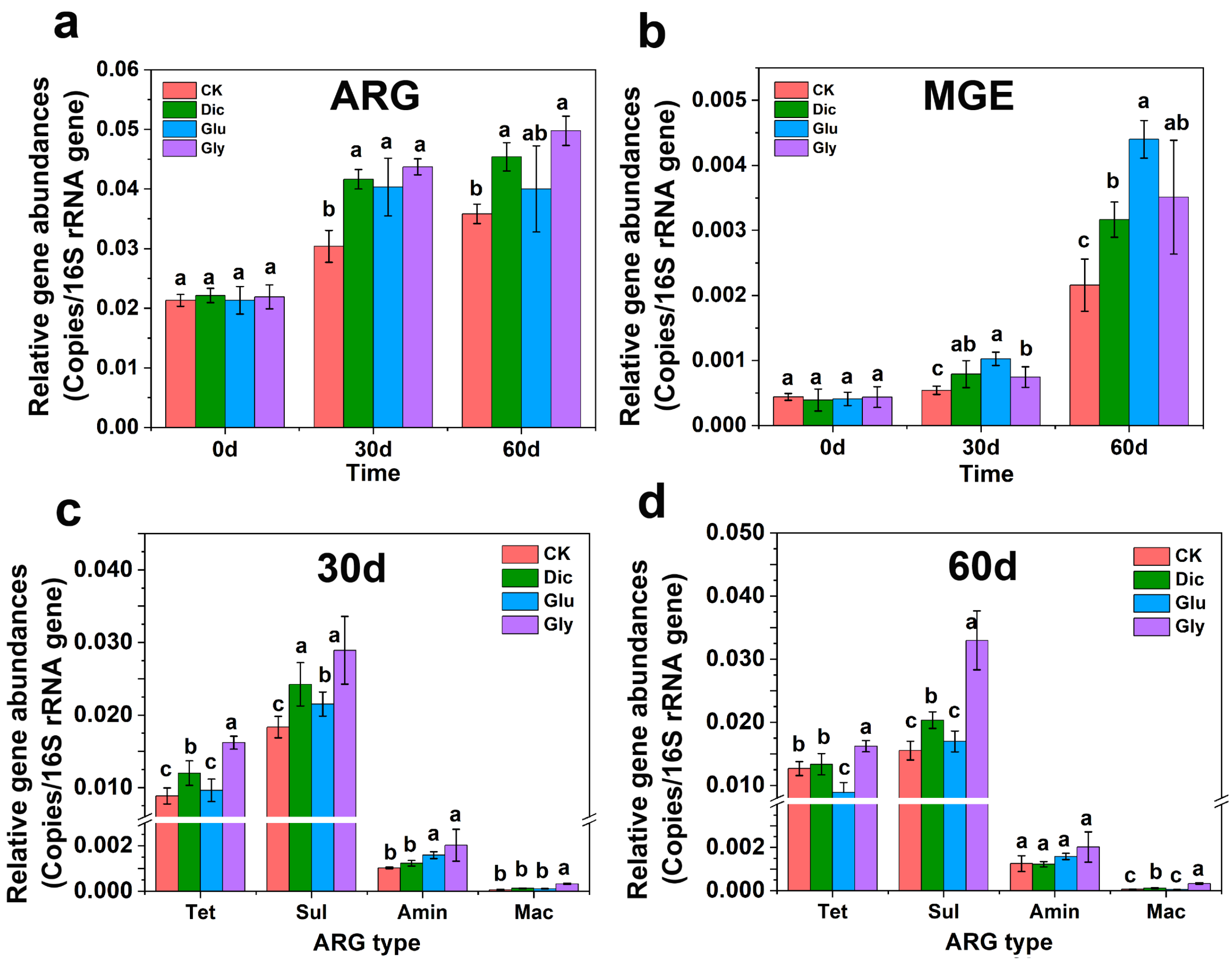

Figure 10. Changes in the relative ARG (a) and MGE (b) abundances in control (CK), glyphosate (Gly), glufosinate (Glu) and dicamba (Dic) treatments at 0d, 30d and 60d sampling time points (based on qPCR analysis). The (c-d) show the changes for different types of ARGs individually in different treatments at 30d (c) and 60d (d) sampling time points (Tet: tetracycline; Sul: sulfonamide; Amin: aminoglycoside; Mac: macrolide). Lowercase letters denote for significant differences between treatments (ANOVA, Bonferroni-adjusted Tukey tests for multiple comparisons, $p < 0.05$). Data show mean ± SD of three biological replicates per sampling time point (n=3 per treatment).

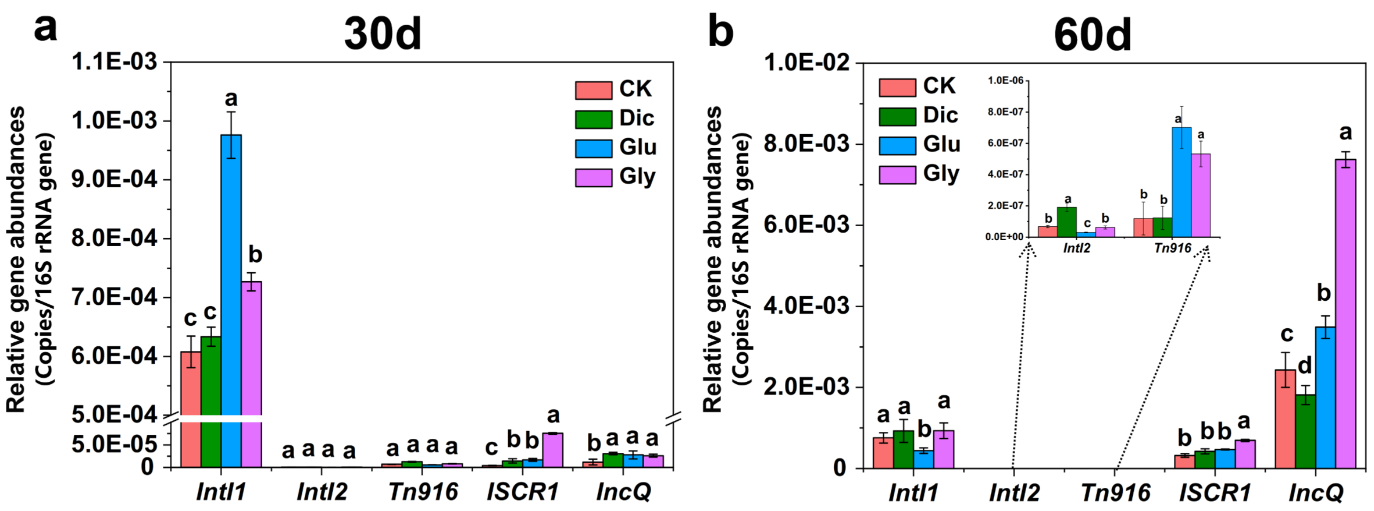

Figure 11. Changes in the relative MGE type abundances (qPCR) in control (CK), glyphosate (Gly), glufosinate (Glu) and dicamba (Dic) treatments at 30d (a) and 60d (b) sampling time points. Lowercase letters denote for significant differences between treatments (ANOVA, Bonferroni-adjusted Tukey tests for multiple comparisons, $p < 0.05$). Data show mean ± SD of three biological replicates per sampling time point (n=3 per treatment).

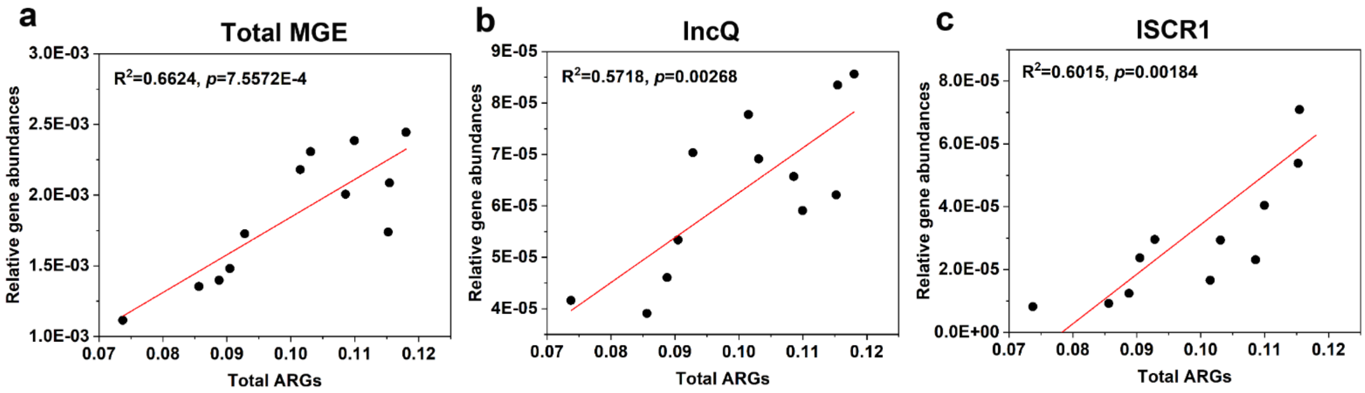

Figure 12. Positive relationship between the total relative ARG abundances and the total relative MGE (a), IncQ (b) and ISCR1 (c) abundances (based on qPCR) over control (CK), dicamba (Dic), glufosinate (Glu) and glyphosate (Gly) treatments (linear regression analysis based on metagenomics data, n=3 per treatment).

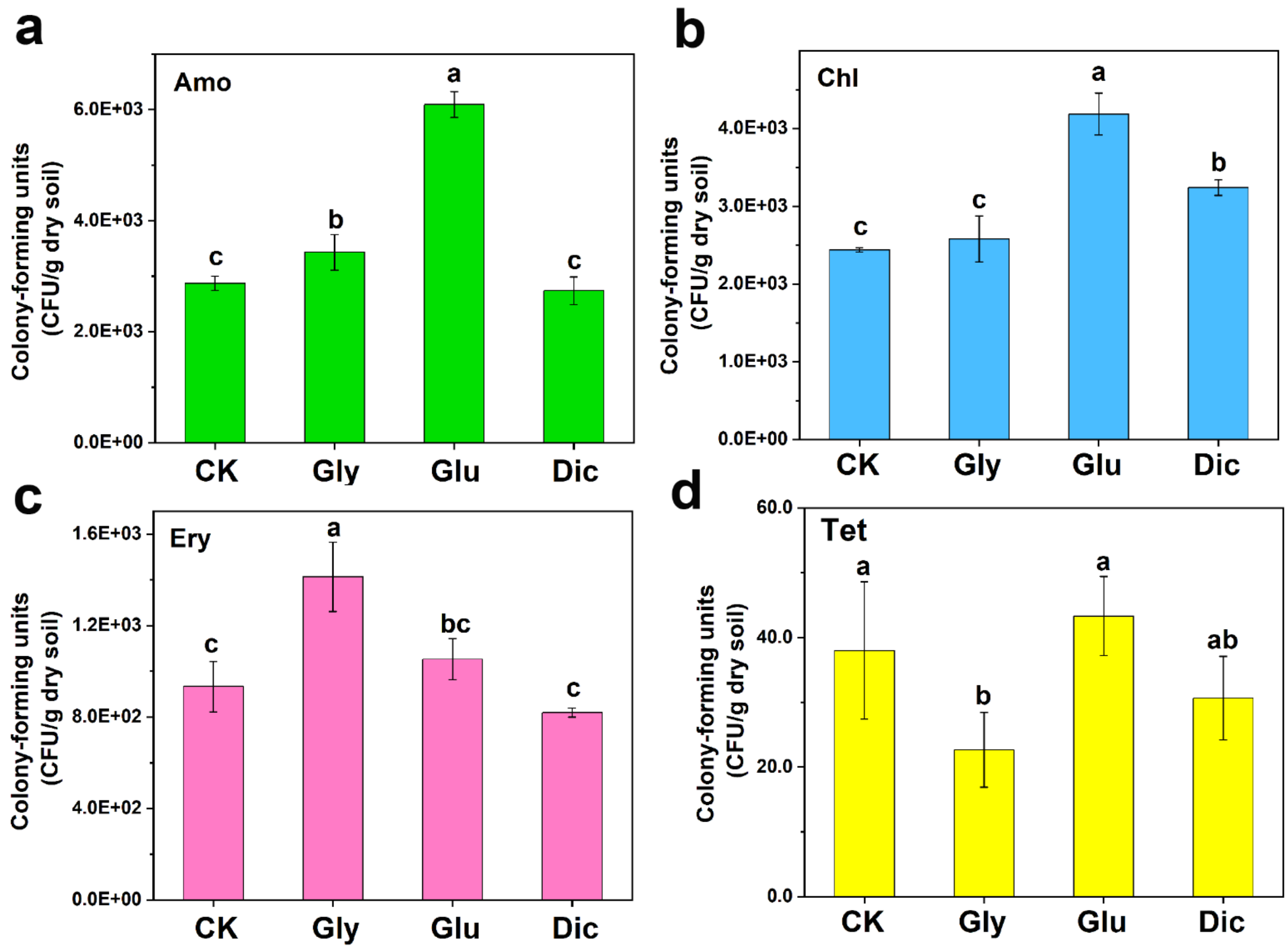

Figure 13. The numbers of culturable antibiotic resistant bacteria (ARB) isolated at 30d time point from control (CK), glyphosate (Gly), glufosinate (Glu) and dicamba (Dic) treatments using selective amoxicillin (Amo, a), chloramphenicol (Chl, b), erythromycin (Ery, c) and tetracycline (Tet, d) antibiotic agar plates. Lowercase letters denote for significant differences between treatments (ANOVA, Bonferroni-adjusted Tukey tests for multiple comparisons, $p < 0.05$). Data show mean ± SD of three biological replicates (n=3 per treatment).

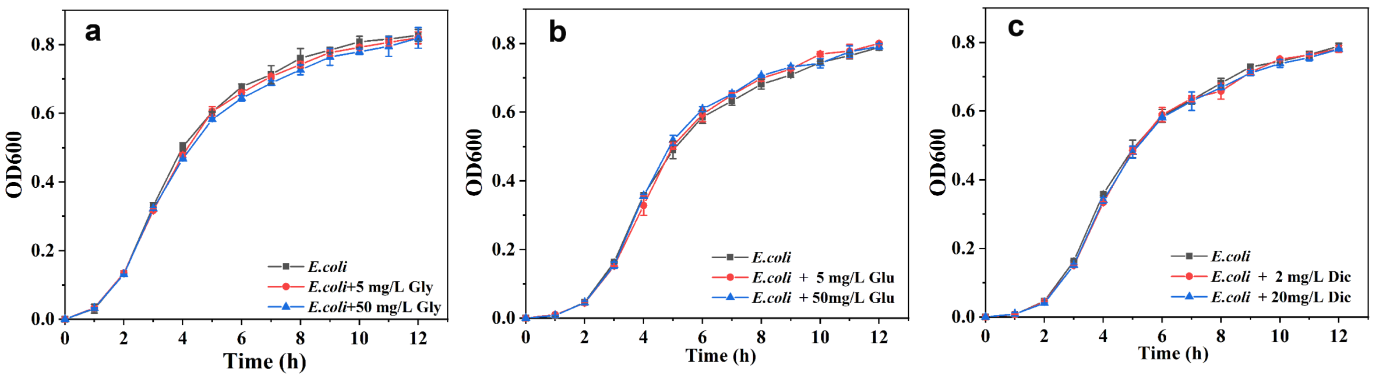

Figure 14. The effect of glyphosate (a), glufosinate (b) and dicamba (c) herbicides on *E. coli* DH5α strain growth in terms of optical density at 600 nm (OD600) at two concentrations (blue and red lines) relative no-herbicide control (black line). Data show mean ± SD of three biological replicates (n=3 per treatment).

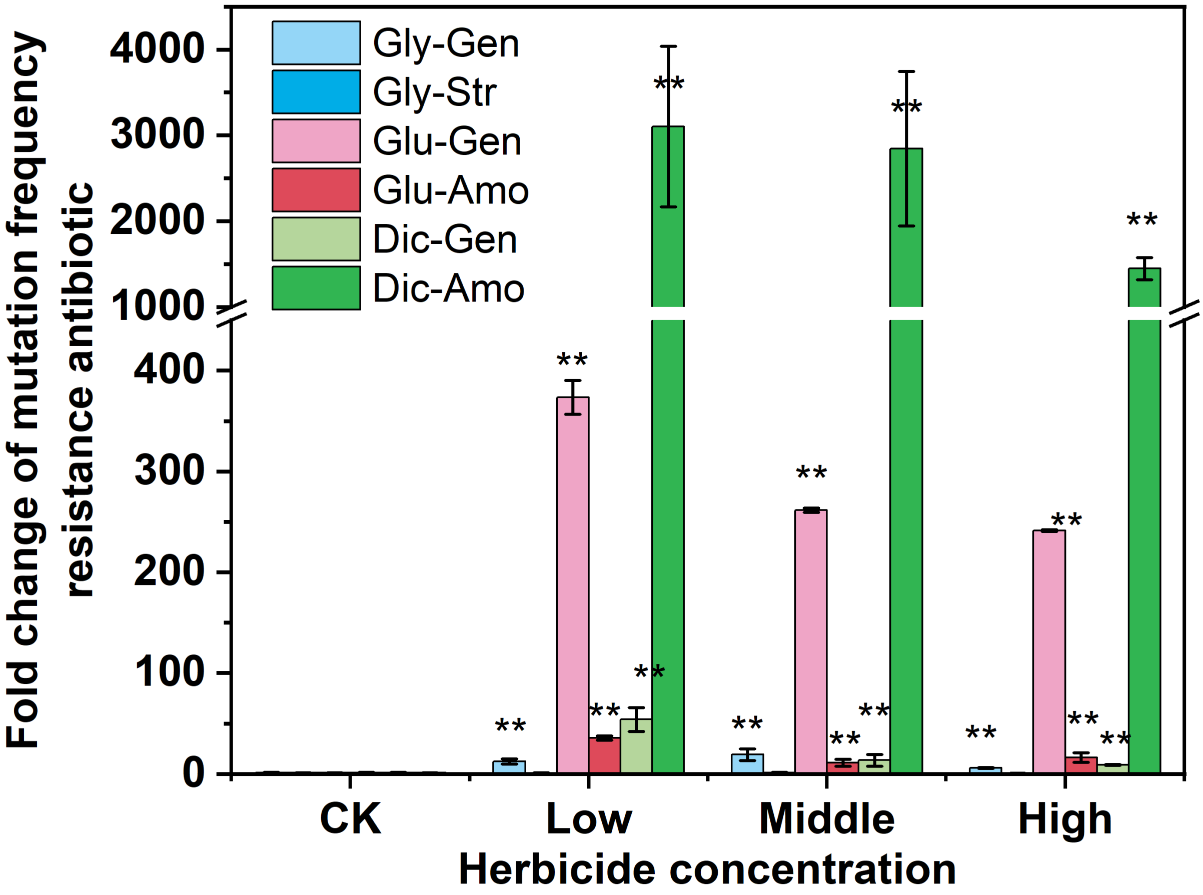

Figure 15. The fold change in the frequency of antibiotic resistant mutants in the absence (CK) or presence of glyphosate (Gly), glufosinate (Glu) and dicamba (Dic) herbicides, in 'low', 'middle' and 'high' concentrations during the selection experiment. The 'low', 'middle' and 'high' herbicide treatments refer to 5, 25, and 50 mg/L concentrations of glyphosate and glufosinate and 2, 10, and 20 mg/L concentration of dicamba, respectively. The fold change in the frequency of antibiotic resistant mutants was calculated based on the sum of mutant frequencies under herbicide relative to no-herbicide treatment. Resistant mutants were screened using gentamycin (Gen), streptomycin (Str) and amoxicillin (Amo) antibiotic agar plates. Data show mean ± SD of three biological replicates (n=3 per treatment).

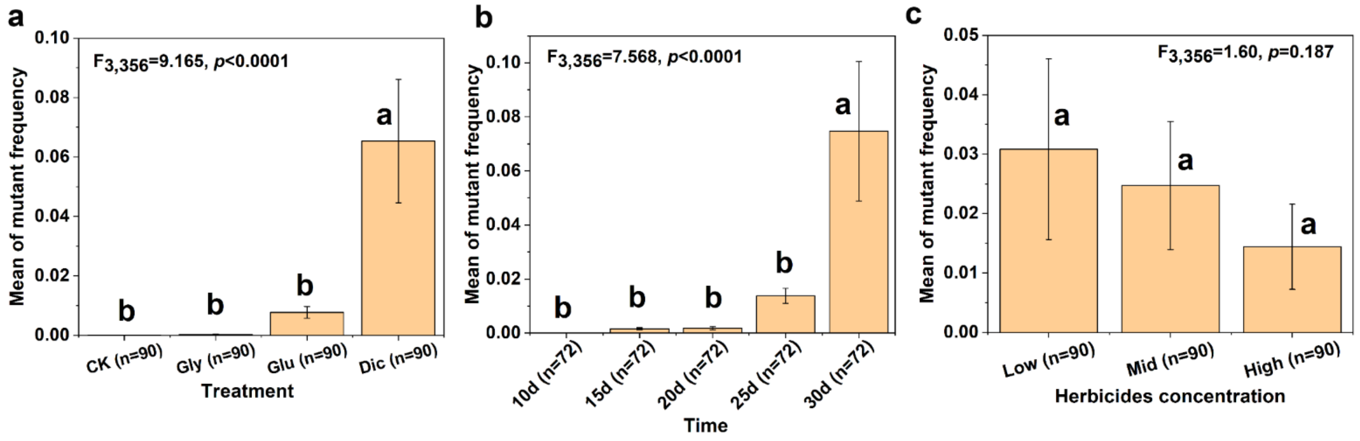

Figure 16. The mean frequency of antibiotic resistant *E. coli* DH5α mutants in the absence (CK) or presence of glyphosate (Gly), glufosinate (Glu) and dicamba (Dic) herbicides (a) averaged across different time points (b) and herbicide concentrations (c). Lowercase letters denote for significant differences between treatments (repeated measures ANOVA, Bonferroni-adjusted Tukey tests for multiple comparisons, $p < 0.05$). Data show mean ± SD of three biological replicates (n=3 per treatment) and the number of clones is shown in parentheses on X-axis.

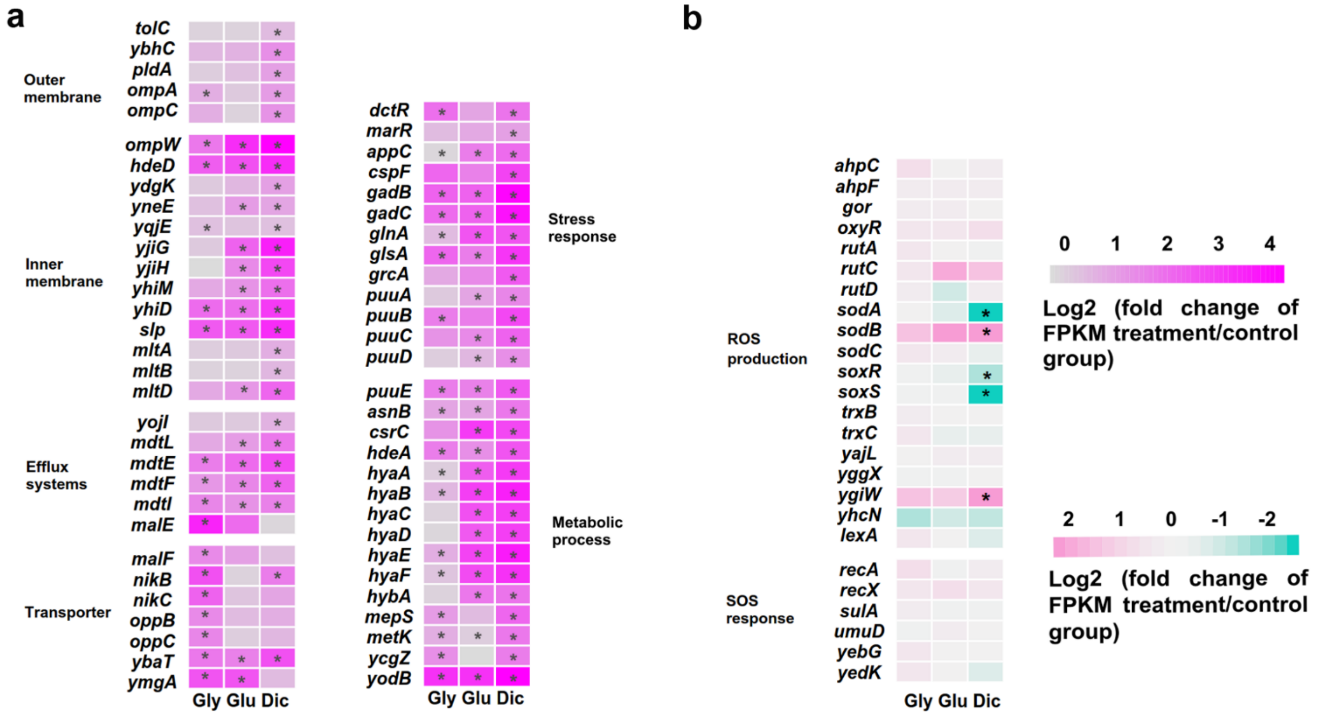

Figure 17. The effect of glyphosate (Gly), glufosinate (Glu) and dicamba (Dic) herbicide exposure on the *E. coli* DH5α ancestral strain gene expression relative to no-herbicide control treatment. Panel (a) shows fold expression changes of core genes related to membrane proteins, efflux systems, transporters, stress responses and metabolic processes potentially associated with antibiotic resistance in *E.coli* DH5α. Panel (b) shows fold expression changes of core genes related to ROS production and SOS response potentially associated with antibiotic or herbicide resistance in *E.coli* DH5α. Significant differences between the herbicide-exposed and control treatment were identified by considering both fold changes ($|\log2FC| > 1$) and adjusted p-values (false-discovery rate corrected $p < 0.05$). All heatmap data show mean ± SD of three biological replicates per treatment (n=3 per treatment).

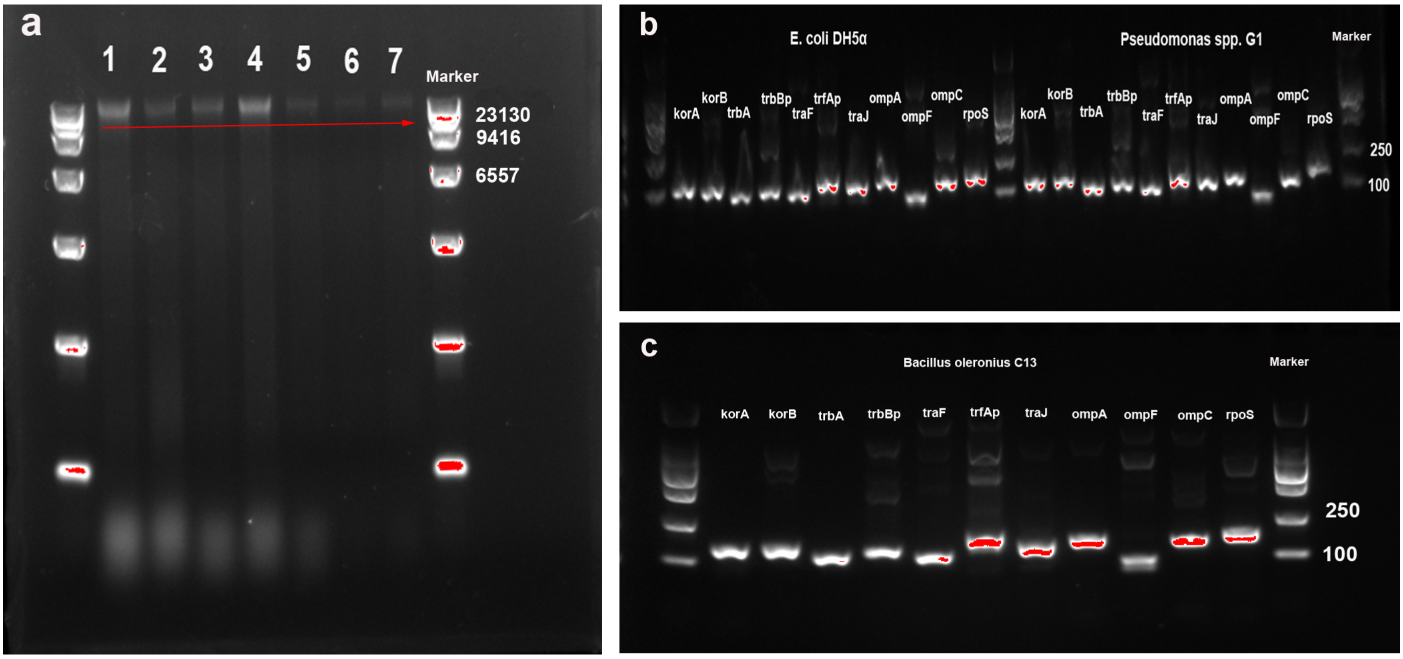

Figure 18. The presence of RP4 plasmid in transconjugants was confirmed using PCR for a subset of clones. (a) shows RP4 plasmid (60099 bp bands visible above red arrow) as follows: lanes 1-2 show plasmids extracted from the donor *E. coli* HB101, lanes 3-4 show plasmids extracted from of *E. coli* DH5α, lanes 5-6 show plasmids extracted from *Pseudomonas* spp. G1 and lanes 6-7 show plasmids extracted from *Bacillus oleronius* C13 transconjugants. (b-c) electrophoresis shows plasmid PCR products for different resistance genes extracted from *E. coli* DH5α, *Pseudomonas* spp. G1 and *Bacillus oleronius* C13 recipient cells.

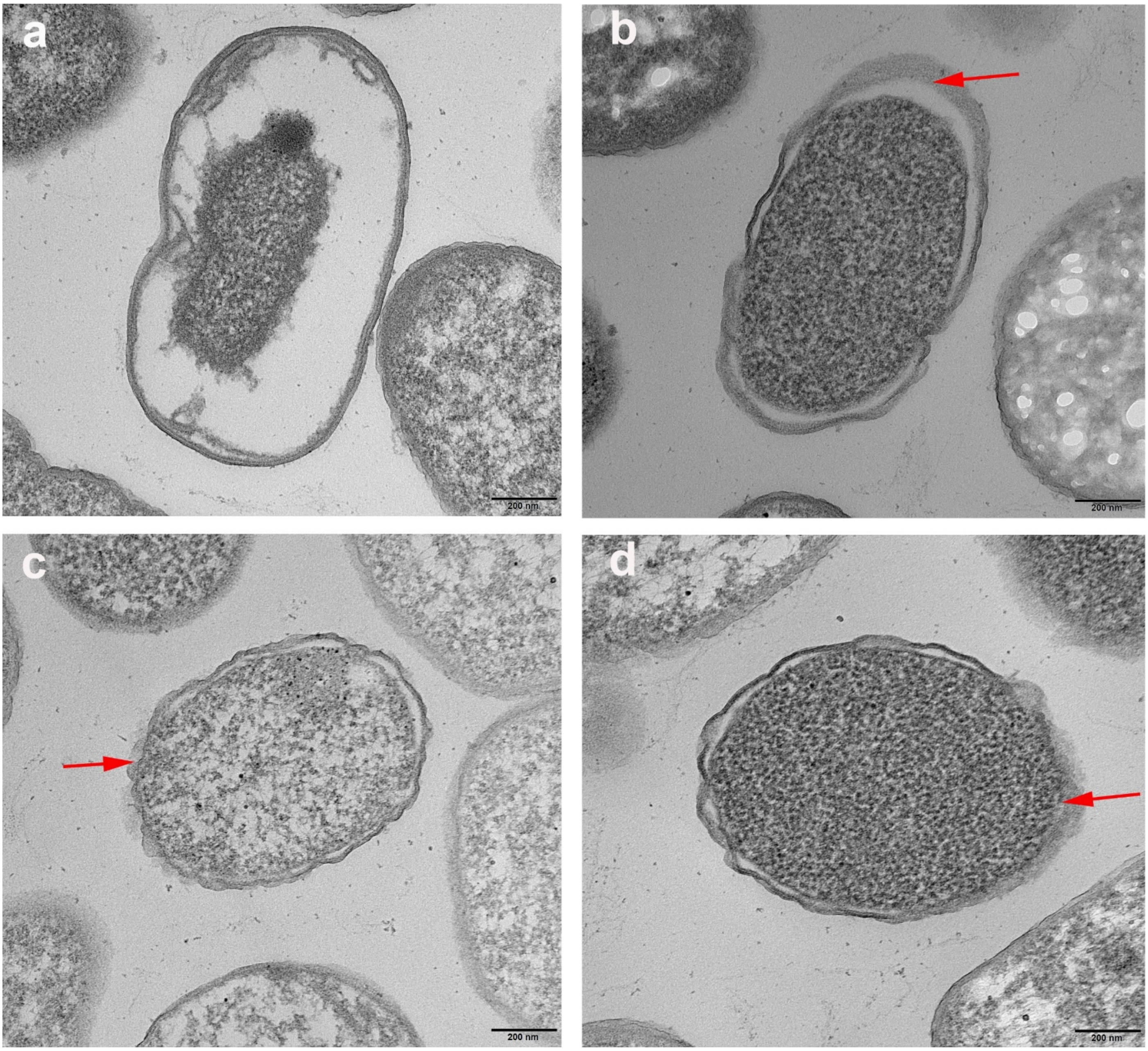

Figure 19. Transmission Electron Microscopy (ultrafine slices) of *E. coli* DH5α cell membranes in no-herbicide control (a) and glyphosate (b), glufosinate (c) and dicamba (d) herbicide treatments (10 mg/L concentration). In (a), the cell membranes are distinct, and the cytoplasm is compact. In contrast, in (b-d), clear cell membrane damage is observed (show in red arrow).

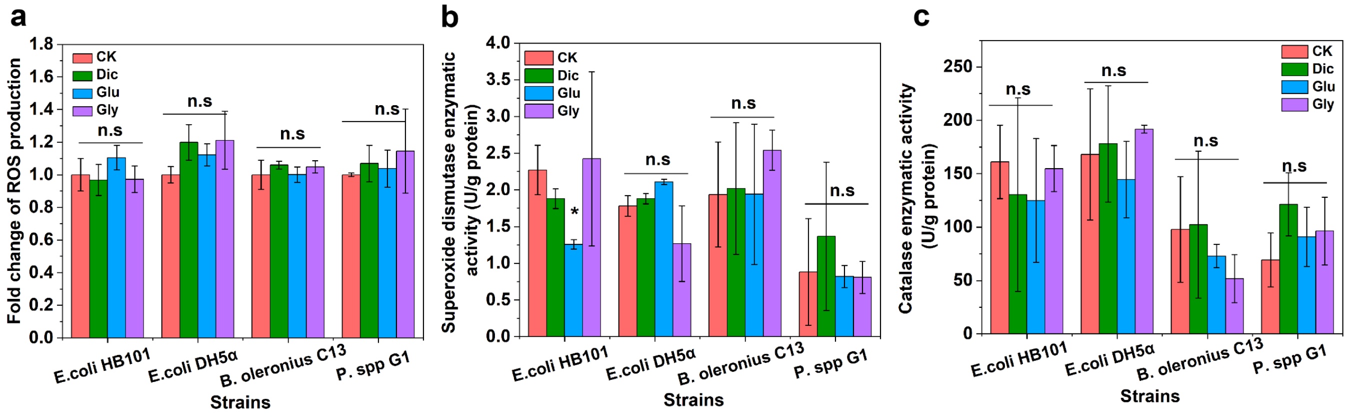

Figure 20. The effect of glyphosate (Gly), glufosinate (Glu) and dicamba (Dic) herbicide exposure (10 mg/L) on ROS production (a) and superoxide dismutase (b) and catalase (c) antioxidant enzymatic activity relative to no-herbicide control (CK) treatment with donor (*E.coli* HB101) and different recipient cells (*E.coli* DH5α; *B. oleronius* C13; *Pseudomonas* spp. G1). Significant differences between treatments were tested by ANOVA and Bonferroni-adjusted Tukey tests for multiple comparisons (* and n.s denote for significant and non-significant differences at p-value of < 0.05, respectively). Data show mean ± SD of three biological replicates (n=3 per treatment).

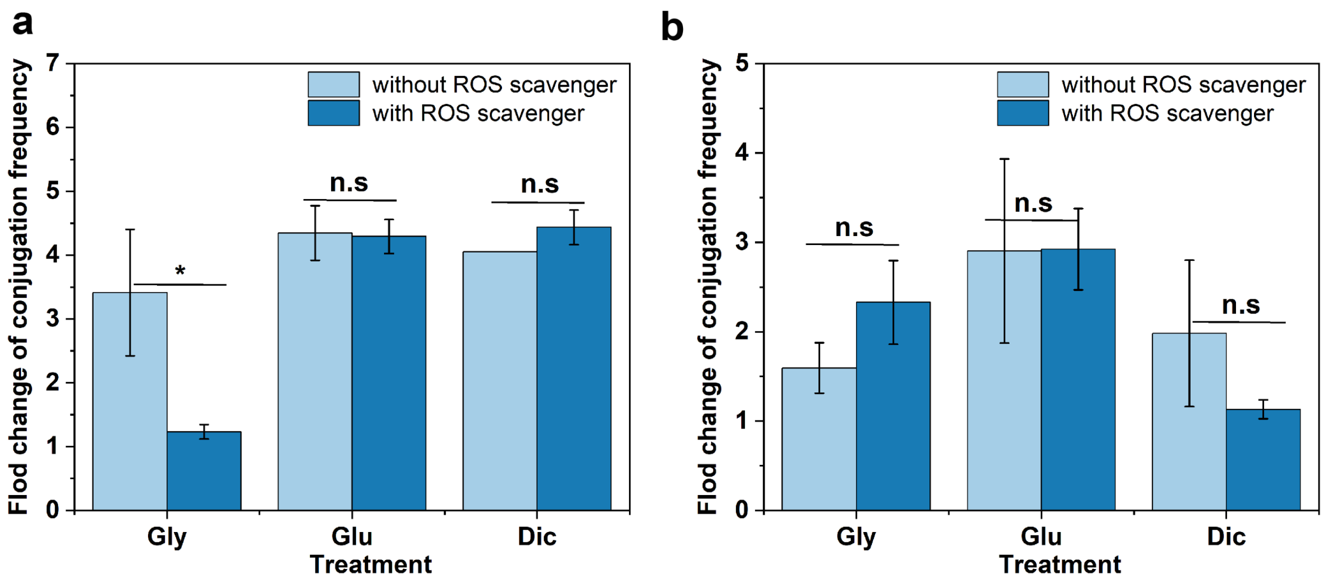

Figure 21. The effect of thiourea ROS-scavenger on the RP4 plasmid conjugation frequency from *E. coli* HB101 strain to *E. coli* DH5α (a) and *Bacillus oleronius* C13 (b) recipient bacterial cells under glyphosate (Gly), glufosinate (Glu) and dicamba (Dic) herbicide exposure (10 mg/L). Significant differences were analysed using independent-sample Student's t-test and *p*-values were corrected by the Benjamini-Hochberg method (* and n.s denote for significant and non-significant differences at *p*-value of < 0.05, respectively. Data show mean ± SD of three biological replicates (n=3 per treatment).

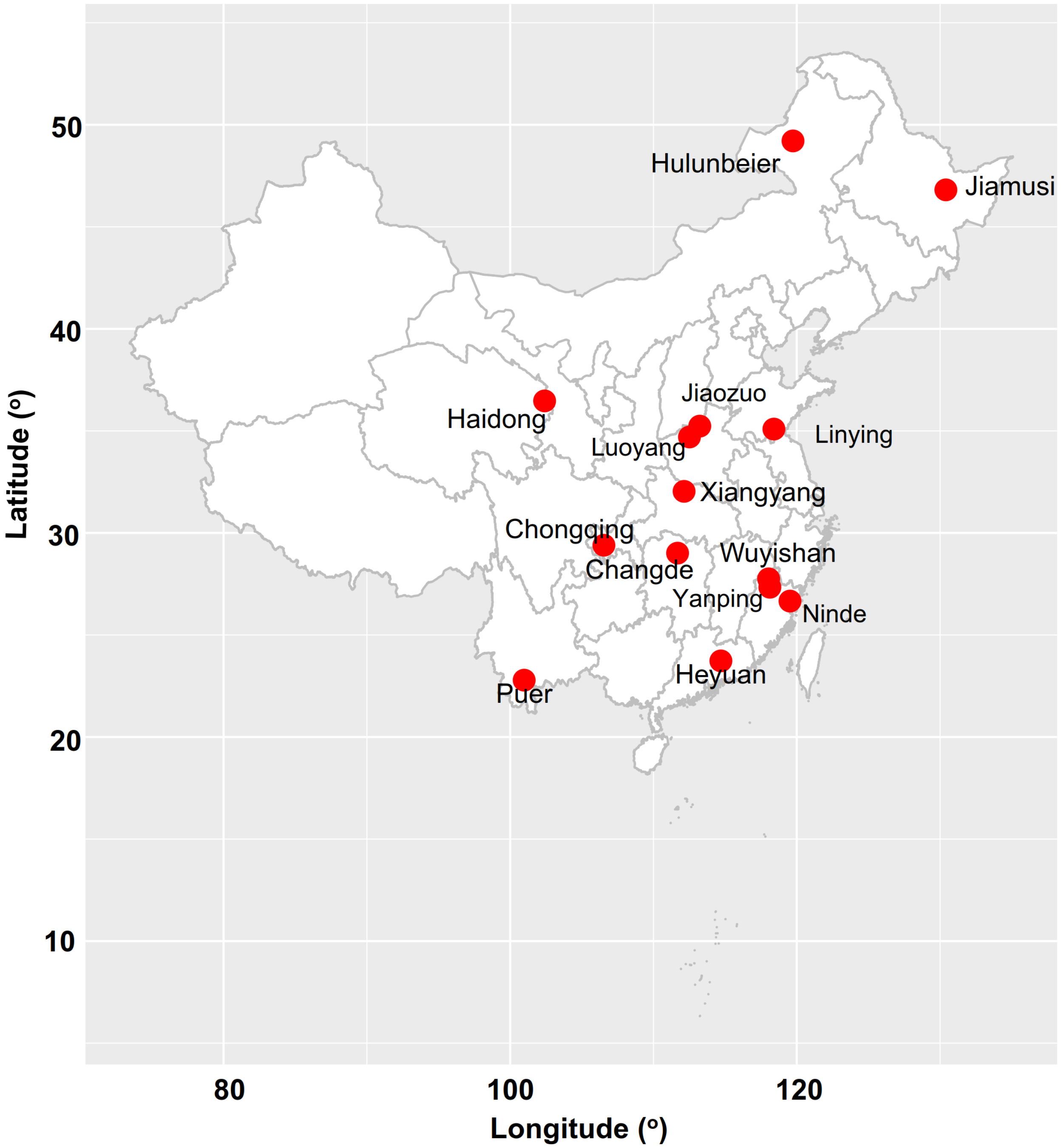

Figure 22. Map showing the location of 21 sampling sites across 11 provinces in China with detailed information on locations and cultivated plants shown supplementary Table S10.

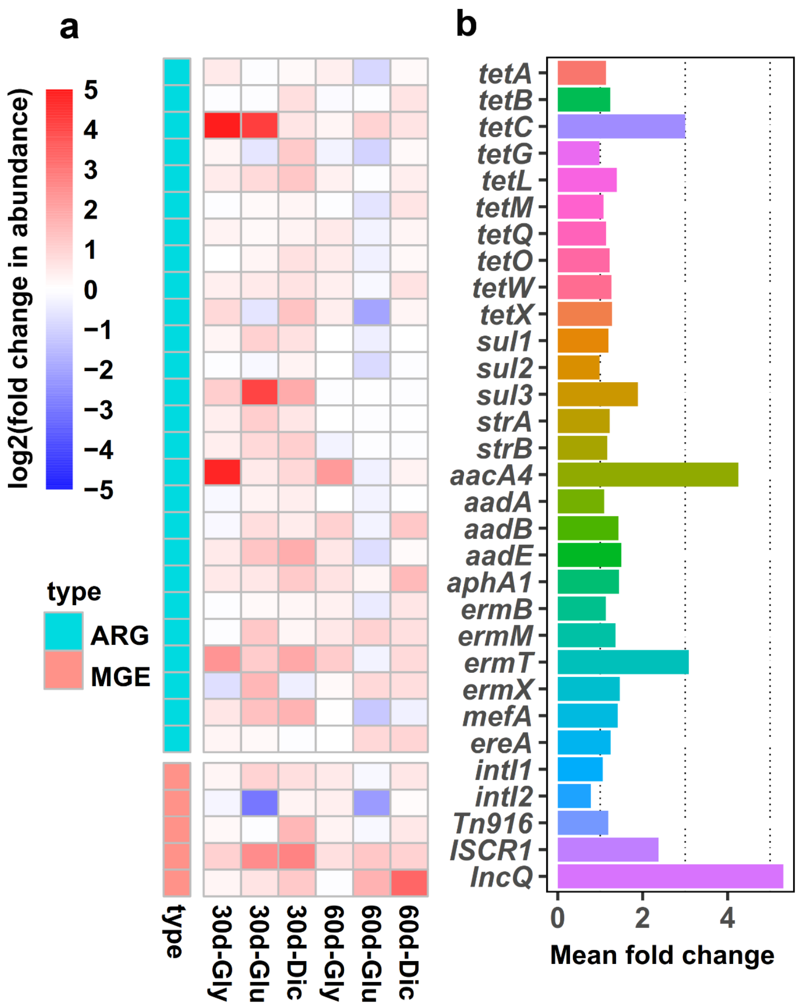

Figure 23. The effect of herbicide exposure on the mean ARG and MGE abundances during the soil microcosm experiment. **(a)** Heatmap shows the fold changes of individual ARG (upper panel) and MGE (lower panel) abundances relative to no-herbicide control treatment. **(b)** The mean target gene abundances across both sampling time points and all herbicide treatments. All data is based on three biological replicates (n=3 per treatment).

References

Druart C, Delhomme O, de Vaufleury A, Ntcho E, Millet M. 2011. Optimization of extraction procedure and chromatographic separation of glyphosate, glufosinate and aminomethylphosphonic acid in soil. Anal Bioanal Chem 399:1725-1732.

Jin M, Liu L, Wang D-n, Yang D, Liu W-l, Yin J, Yang Z-w, Wang H-r, Qiu Z-g, Shen Z-q, et al. 2020. Chlorine disinfection promotes the exchange of antibiotic resistance genes across bacterial genera by natural transformation. The ISME Journal 14:1847–1856.

Liao H, Lu X, Rensing C, Friman VP, Geisen S, Chen Z, Yu Z, Wei Z, Zhou S, Zhu Y. 2018. Hyperthermophilic composting accelerates the removal of antibiotic resistance genes and mobile genetic elements in sewage sludge. Environmental Science & Technology 52:266-276.

Nayfach S, Pollard KS. 2015. Average genome size estimation improves comparative metagenomics and sheds light on the functional ecology of the human microbiome. Genome Biology 16:51.

Parnanen K, Karkman A, Hultman J, Lyra C, Bengtsson-Palme J, Larsson DGJ, Rautava S, Isolauri E, Salminen S, Kumar H, et al. 2018. Maternal gut and breast milk microbiota affect infant gut antibiotic resistome and mobile genetic elements. Nature Communications 9:3891.

Shin EH, Choi JH, Abd El-Aty AM, Khay S, Kim SJ, Im MH, Kwon CH, Shim JH. 2011. Simultaneous determination of three acidic herbicide residues in food crops using HPLC and confirmation via LC-MS/MS. Biomedical Chromatography 25:124-135.

Yin X, Jiang X-T, Chai B, Li L, Yang Y, Cole JR, Tiedje JM, Zhang T, Wren J. 2018. ARGs-OAP v2.0 with an expanded SARG database and Hidden Markov Models for enhancement characterization and quantification of antibiotic resistance genes in environmental metagenomes. Bioinformatics 34:2263-2270.

549
